# Modulation of miRNA expression in natural populations of *A*. *thaliana* along a wide altitudinal gradient of Indian Himalayas

**DOI:** 10.1038/s41598-018-37465-y

**Published:** 2019-01-24

**Authors:** Abhinandan Mani Tripathi, Akanksha Singh, Rajneesh Singh, Ashwani Kumar Verma, Sribash Roy

**Affiliations:** 1 Division of Molecular Biology and Biotechnology, CSIR-National Botanical Research Institute, Rana Pratap Marg, Lucknow, Uttar Pradesh India; 2 Academy of Scientific and Innovative Research (AcSIR), Anusandhan Bhawan, 2 Rafi Marg, New Delhi, India

## Abstract

Plant populations growing along an altitudinal gradient are exposed to different environmental conditions. They are excellent resources to study regulatory mechanisms adopted by plants to respond to different environmental stresses. Regulation by miRNA is one of such strategies. Here, we report how different miRNAs are preferentially expressed in the three natural populations of *A*. *thaliana* originating from a wide altitudinal range. The expression level of miRNAs was mostly governed by temperature and radiation. Majority of the identified miRNAs expressed commonly in the three populations. However, 30 miRNAs expressed significantly at different level between the low and the high altitude populations. Most of these miRNAs regulate the genes associated with different developmental processes, abiotic stresses including UV, cold, secondary metabolites, etc. Further, the expression of miR397 and miR858 involved in lignin biosynthesis and regulation of secondary metabolites respectively, may be regulated by light intensity. A few miRNAs expressed at increasing level with the increase in the altitude of the site indicating environment driven tight regulation of these miRNAs. Further, several novel miRNAs and isomiR diversity specific to the Himalayas are reported which might have an adaptive advantage. To the best of our knowledge, this is the first report on miRNA expression from natural plant populations.

## Introduction

Plants growing under natural conditions are exposed to varying environmental stresses^1,2^. Being sessile, they have evolved several strategies to cope with such biotic and abiotic stresses. Understanding the mechanisms of abiotic stress tolerance and/or avoidance is a central goal for plant biologists. One of such strategies deployed by plants is the regulation by micro RNA (miRNA). The role of miRNA to cope with various stresses have well been described in various organisms^3–8^. The miRNAs are a class of small RNA (21–24 nt long) that modulates gene expression either at the post-transcriptional level by degrading mRNA or at the translational level by blocking protein biosynthesis at different stages^9–11^. They are processed from their precursors by the DCL1 enzyme and, the mature (guide) strand of miRNA is incorporated in the AGO1 complex to target the mRNA. The abundance of mature miRNAs depends upon the miRNA types and the environmental stimulus^5,12^. For example, most of the conserved miRNAs like miR156, miR166, and others are expressed at a higher level than non-conserved miRNAs like miR775, miR8183 and others^6,13^. Recent advancement in next-generation sequencing techniques and bioinformatics has paved the way for large-scale discovery and identification of miRNAs in different plant species^14–18^.

Several recent studies have firmly established that the level of miRNA expression in plants is dynamically regulated in response to different stresses^3,5,7,8,19–21^. Involvement of miRNAs in response to environmental stresses, including drought^22–25^, salinity^25–27^, high temperature^3^, low temperature^15^, UV light^7,28^, high or low light intensity^29^, hypoxia^30,31^, heavy metal^5,32^, etc have been reported. A total of 1511 miRNAs are known to be involved in various abiotic stresses in different plant species^33^. In the case of *Arabidopsis*, miR156, miR159, miR167, miR168, miR171, miR172, miR319, miR393, miR394a, miR395c, miR395e, miR396 and miR397 are up-regulated, while miR161, miR168a, miR168b, miR169, miR171a, and miR319c are down-regulated, under drought stress^8,34^. While other miRNAs such as miR396, miR168, miR167, miR165, miR319, miR159, miR394, miR156, miR393, miR171, miR158, and miR169 were altered in response to salt stress^8,25^ and miR393, miR397b, miR402 and miR319c expression were up-regulated during cold stress^35^. Similarly, miR858 have been shown to be regulated by light^36^. Further, genotype-specific expression of miRNAs under abiotic stress, for example in the cold has been reported^16,37^.

However, most of these studies were conducted by application of a specific type of abiotic stress treatment under controlled condition. While these and other studies provided insight into the miRNA regulation, report on their regulatory role under field condition is scanty. Since under natural condition plants are exposed to a combination of various climatic, edaphic and other biotic factors^1^ plants response towards such abiotic stresses, and thus miRNA expression may differ. From the evolutionary point of view, populations growing under different climatic conditions may have adopted different strategies to cope with the prevailing climatic conditions. It has also been reported that some miRNAs emerge de-novo in response to evolutionary adaptation, driven by positive selection^38^. Besides these canonical miRNAs, the other non-canonical (isomiRs) forms of mature miRNAs and their role are poorly understood. It has been shown that the expression of isomiRs varies among different tissues and under different pathological conditions^39–41^. They also interact cooperatively with canonical miRNA sequences that help in targeting common pathways^42,43^.

Plant populations growing along a wide altitudinal gradient are excellent resources to study the influence of environment on various molecular, physiological, biochemical and other developmental processes^44^. This is because there are large ecological and environmental variations within a short spatial distance. Populations at high altitude face extreme environmental conditions like high UV radiation, low temperature, high light intensity, low oxygen availability, large variations in the day-night temperature, etc. as compared to the lower altitude populations. Such plants have also evolved strategies to counter such stresses. For examples, the presence of shorter leaves with higher phenolic contents to overcome the effect of UV light and low temperature^45,46^, over-accumulation of osmolytes and polysaccharides in response to cold^47^ etc. Thus, the plants growing at high altitude might require a more complex network of regulation to cope with the various abiotic stresses as compared to the lower altitude populations.

*A*. *thaliana* is an established model plant species for deciphering molecular and ecological adaptations owing to its world-wide distribution and natural genetic variations^48–50^. *A*. *thaliana* populations of Indian West Himalaya inhabit a unique mountainous habitat which ranges from subtropical to a temperate climate. These populations follow a summer-annual life history. Previous studies showed that these populations are distinct from each other at least at altitudinal level^51,52^. Tyagi *et al*. (2016) reported that the high light intensity prevalent in the high altitude area might play a critical role in the emergence of population-level variations in these populations^53^. Thus these populations may be the valuable resources to study the mechanisms of miRNA regulation to cope with different environmental stresses.

In the present study, using small RNA sequencing we aimed to find out the expression pattern of miRNA in the three natural populations of *A*. *thaliana* originating from the three different altitudes, designated as low (700 m asl), medium (2000 m asl) and high altitude (3400 m asl) populations. Since these populations originated from a wide altitudinal range, they are exposed to different environmental conditions, and hence the miRNA expression pattern will be different in the three populations. This may provide clues for the regulatory and adaptive role of different miRNAs in real time. Further, since these populations were never studied with respect to miRNA, it may provide novel miRNAs with adaptive significance. We also compared the miRNA expression pattern of the field populations to their controlled condition grown counterpart to highlight the influence of environmental factors on the miRNA expression pattern. We identified a few novel miRNAs and population-specific miRNAs. Finally, we report the variation in miRNA arm expression (5p/3p), isomiR diversity and their canonical and non-canonical forms. This is the first report explaining the miRNA expression from natural populations of *A*. *thaliana* from the habitat of widest known altitudinal range.

## Material and Methods

### Collection of samples

The details of the collection sites, population habitat, and collection of samples were as described earlier in Tyagi *et al*.^53^. Briefly, leaf samples of *A*. *thaliana* plants were collected from three different populations in the West Himalaya. The samples were collected from each population for the two consecutive years of 2013 and 2014 from the field (FD) at a stage defined as plants with the first flower open (principal growth stage 6.00)^54^ to plants having no more than ten green siliques. To minimize the effect of the microclimatic variables, the areas in each site was selected which maximally represented plants of almost uniform growth and exposed to sunlight. From five such distinct patches, a total of 50 individuals were randomly selected for each population. A minimum distance of two meters was maintained between any two individuals to minimize the collection of single-parent descended siblings. One to two rosette leaves were collected from each plant. The leaves were cut into smaller pieces and stored in the RNAlater™ solution (Ambion, USA) at 4 °C till these were brought back to the laboratory.

The seeds from mature plants were also collected from each site. These seeds were grown under controlled conditions (GH) of 22 °C temperature, 140 µmol/m^2^/sec light intensity and 16/8-hour day/night cycle. Additionally, the seeds of the *A*. *thaliana* ecotype, Col-0 have also been grown as a control for the glasshouse grown plants. The leaf tissue of GH plants was collected at a stage similar to that of FD (Supporting Information Figure S1). To minimize sampling variation of the GH samples with those of the field, samples were stored in RNAlater™ solution at 4 °C for 24 hours before the extraction of total RNA.

### Extraction of total RNA and sequencing

Total RNA was extracted from the leaf tissue of ten plants representing one patch from the FD as well as from the GH grown plants. The RNAlater™ preserved leaves were washed with Milli-Q water, blotted with a tissue paper and weighed. The equal amount of tissues were used for extraction of total RNA by using the mirVana™ miRNA/total RNA isolation kit (Ambion, USA), following the manufacturer’s protocol. The quantity, quality and RNA Integrity Number (RIN score) of the total RNA were determined by QIAxpert™ microfluidic UV/VIS spectrophotometer (Qiagen, USA) and 2100 BioAnalyzer™ (Agilent Technologies, USA), respectively. Equal amounts of high-quality RNA from each patch of a population were pooled together (total five patches) and considered as representative of the respective population. The pooled RNA was treated with DNase (Ambion TURBO DNA-free DNase kit) according to the manufacturer’s instructions. A total of 10 Barcoded cDNA libraries, three each from FD pooled samples from the year 2103 and 2014, three from the GH plants and one of Col-0 were prepared using the TruSeq™ RNA Sample Prep Kit v2. (Illumina, USA) with one microgram of total RNA as input, as per the manufacturer’s protocol. Single-end sequencing of these libraries was performed in a single lane using the Illumina HiSeq1000 platform.

### Pre-processing of raw sequencing reads and identification of known miRNAs

Ten small RNA library sequences were generated from high throughput sequencing. The adapter, low quality and sRNA (18–28 nt) reads were filtered simultaneously by using adapter removal tool in UAE small RNA workbench^55^. The size-filtered reads were mapped on *A*. *thaliana* reference genome (TAIR10) implemented in UAE small RNA workbench. The genome mapped reads were extracted and mapped sequentially on *A*. *thaliana* cDNA (TAIR10), t/rRNA (http://rfam.xfam.org/), sno/sn RNA (http://rfam.xfam.org/) and tasi-RNA (http://bioinfo.jit.edu.cn/tasiRNADatabase/). The reads mapped on above RNAs were discarded, and unmapped reads were now called as cleaned reads. These cleaned reads were mapped again on mature miRNA sequences of *A*. *thaliana* reported in miRBAse21^56^. Finally, these mapped reads were identified as miRNA and used for differential expression analysis. The clean reads that did not map with miRBase 21 sequences were used for novel miRNAs prediction. All of the above analysis was performed using UAE small RNA workbench^55^.

The secondary structures of putative novel miRNAs were predicted by miRCat^55^ using default parameters (except the minimum read abundance was 10). The predicted secondary structures were analyzed manually by using following parameters: 1) The miRNA and miRNA* are derived from opposite stem arms such that they form a duplex with two nucleotide 3′ overhang; 2) base-pairing between the miRNA and miRNA* is extensive such that there are typically four or fewer mismatches; 3) The frequency of asymmetric bulges is one or zero and size of the bulges is no more than two nucleotide within miRNA duplex. The detailed pipeline of deep sequencing data analysis is shown in (Supporting Information Figure S2). All sequencing data generated has been deposited in National Center for Biotechnology Information (NCBI) with sequence read archive (SRA) accession # SRP140816.

### Differential expression analysis

The read counts of each miRNA were normalized to reads per million (RPM). If original miRNA expression in a library was zero, the normalized expression was adjusted to 0.01according to a previous report^57^. Log2 fold changes in miRNA expression were calculated between the two libraries only when read count were more than 10 in any one of the libraries. A 2 × 2 contingency table was used to perform Pearson’s chi-square test of significance for variable expression between the two libraries^3^. We defined uniquely expressed miRNAs as miRNAs expressed only in one population and not in the other two populations. Environment-specific miRNAs were identified using two-way ANOVA where, population (Pop), growing condition (TMT) and their interaction (Pop x TMT) were used as factors. We also compared the relative expression level of the 5p and 3p arms of the miRNA duplex in each population. Only those miRNAs showing expression of both the arms in all the populations were analyzed, and the ratio of 5p and 3p were compared both within and between the three populations. To represent the diversity of miRNA, Jaccard similarity index was calculated using R package DIVO.

### Expression analysis of isomiRs

The cleaned reads were mapped on annotated *A*. *thaliana* mature miRNAs and their precursors. The mapped reads were extracted and the read counts were normalized using the same approach as applied for total miRNAs. We categorized mapped reads into seven categories: [1] Canonical sequences- exact mature sequences available in miRBase21, [2] shifted isomiRs- sequences is shifted towards 5′ or 3′ on precursor with respect to canonical miRNA sequence, [3] substitution isomiRs- central mismatches in small RNA with respect to canonical miRNAs 4] both end variation isomiRs- sequence varies at both ends of small RNA, [5] 3′- template isomiRs- variation at 3′-ends either deletion or addition of bases, 6] 3′-non template isomiRs- variation at 3′ end with extra-base added and [7] start site variation isomiRs- variation at 5′ start sites. The isomiR diversity was calculated as the ratio of expression of dominant isomiR to total expression of the miRNA^41^.

### miRNA target and their Gene Ontology categories

Targets of differentially expressed miRNAs were predicted using the psRNA target on *A*. *thaliana* transcript (TAIR10) using default parameters. Expressions of the predicted targets were evaluated using previously reported transcriptome data of the same samples (accession # SRA347035)^53^. The normalized reads of the transcript as described for miRNAs were used for differential expression analysis. The log2 fold change of miRNA expression and its corresponding target gene expression were compared. The biological function of miRNA targets was analyzed using agriGO against *A*. *thaliana* genome (TAIR 10) as background. The GO terms were considered to be enriched when FDR value was less than 0.05. For further reduction of the redundant GO terms, REViGO (http://revigo.irb.hr/) analysis was performed using default parameter settings, and clustering patterns of the GO terms (with FDR < 0.05) were obtained.

### Real-time quantification of miRNAs

Differentially expressed miRNAs identified by deep sequencing were further validated by stem-loop quantitative real-time PCR^58^. The stem-loop PCR is a reliable method for detection and quantification of miRNAs. For validation, 16 miRNAs were randomly selected which expressed differentially between any two populations in the field condition. The primer sequences are listed in Supporting Information Table S1. For each comparison, three biological and two technical replicates were considered. Individual libraries were prepared for each miRNA using 500 ng of total RNA. cDNAs were synthesized from the stem-loop by using SuperScriptIII reverse transcriptase (Invitrogen, USA). To check the expression level, 2 ul cDNAs were amplified by using miRNAs specific forward primer and stem-loop specific reverse primer. qRT-PCR was performed with DyNAmo Flash SYBR Green (Thermo Fisher, USA) with cycling conditions as follows: denaturation at 95 °C for 10 min, followed by 40 cycles of denaturation at 95 °C for the 20 s, annealing, and extension together at 60 °C for the 60 s. The amplification reaction was performed using ABI7300 real-time PCR system (Applied Biosystems). 5S rRNA gene was taken as an endogenous control for miRNAs expression analysis^3^.

### Climatic data analysis

The 19 bioclimatic variables of three different altitudinal ranges were collected from WorldClim database^59^ at the highest available resolution of 30” (second). Data for solar radiation was extracted from CliMond database^60^ at the highest available resolution of 10′ (minute) (Supporting Information Table S2). As the miRNA expression is primarily governed by the environmental condition prevailing during the growing period of the plant, three additional bio-climatic factors viz. average temperature (AT), average precipitation (AP) and average radiation (AR) prevailing during the growing season of the respective populations were also derived as reported earlier^53^. The principal component analysis was performed for these 22 bioclimatic variables to classify the three sites by multivariate climatic associations and reducing the number of factors. The principal component score of the significant component axis was calculated, and Pearson’s correlation was performed with the normalized reads of commonly expressed miRNAs. This led us to identify miRNA that was influenced by the overall climatic variation of the site. Further, to identify the association of miRNA with specific environmental factor another Pearson’s correlation analysis was performed with the derived bioclimatic factor of the growing season.

### UV-B treatment

A total of 90 plants from the three populations (5 accessions × 3 populations × 3 replicates × 2 conditions) were grown in plastic pots filled with Soilrite^®^ after four days of stratification at 4 °C. The plants were grown for 21 days in GH under 16-/8-h photoperiods. After 21 days, 15 pots from each population were transferred to photo-chamber with 16-/8-h photoperiods and 40 μmol m^−2^ s^−1^ UV-B light (TL20W/01RS, Philips) for seven days. After seven days of UV treatment, leaf traits and dry weight of plants were measured. The data were analyzed in R using 2-way ANOVA with population, UV treatment and their interaction as factors.

### Lignin quantification

Differential accumulation of lignin in stems of FD and GH grown plants of the same stage were visualized through transverse section of stem stained with 1% aqueous Astra-blue (Hi-media, India) followed by 1% aqueous Safranin (Hi-media) under the microscope (Leica Microsystems, Germany). Total lignin content was estimated following Bruce and West (1989). Briefly, 600 mg stem samples were crushed in liquid nitrogen by using mortar and pestle. The crushed samples were dissolved in 2 ml ethanol and centrifuged at 12000 rpm for 30 min at 4 °C. The pellet was dried at 25 °C for 12 hrs and dissolved in 5 ml HCl (2 N) and 0.5 ml thioglycolic acid solution followed by incubation at 95 °C for 8 hours. The dissolved samples were centrifuged at 12000 rpm for 30 min. The resulting pellet was washed with 2.5 ml double distilled water and centrifuged at 12000 rpm for five minutes. The pellet was dissolved in 5 ml NaOH (1 N) at 25 °C overnight with gentle agitation followed by centrifugation at 12000 rpm for 30 min at 4 °C. The resulting supernatant was mixed with 1 ml of HCL (2 N) and precipitated overnight at 40 °C. The sample was centrifuged at 12000 rpm for 30 min at 4 °C and resulting pellet was dissolved in 3 ml NaOH (1 N). The absorbance of the resulting solution was measured at 280 nm.

### Accession codes

The raw data has been submitted to NCBI under accession # SRP140816.

## Results

### Analysis of small RNAs

**Figure 1:**
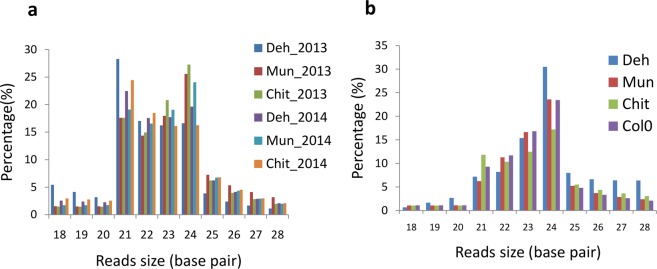
Histogram showing length distribution of small RNA sequence from (**a**) field and (**b**) glasshouse samples.

Leaf samples were collected from the three populations in the year 2013 and 2014. Small RNA sequencing of FD samples yielded 15 to 20 million and 23 to 30 million raw reads in the year 2013 and 2014, respectively. On the other hand, 22 to 28 million raw reads were obtained from GH grown plants. After adapter removal, quality control and size filtering (18–28 nt) of the reads, about 70% to 80% of remaining reads mapped to *A*. *thaliana* genome. After removal of other contaminating RNAs, on an average 3861285 and 4096769 cleaned reads were obtained from 2013 and 2014 FD data sets, respectively while 3411313 cleaned reads were obtained from GH datasets (Supporting Information Table S3). The length distribution of the sRNA ranged from 18–28 nucleotides. The distribution pattern of sRNA slightly varied between FD and GH. In FD samples, the 21 and 24-nucleotide sized groups were the dominant one followed by 23 and 22 nucleotide size and in GH samples the 24-nucleotide sized reads were the major ones in all the samples. The read length distribution and its pattern were consistent for both the years (Fig. 1).

### Identification of known miRNAs

All the clean sRNA reads from different libraries were pooled together and mapped against miRBase21 to identify miRNAs. A total of 252 miRNAs belonging to 78 families were identified from the pooled data having at least ten reads for each miRNA. Following same criteria, a total of 128, 151, 177 miRNAs were identified from the FD samples of Deh, Mun, Chit, respectively in the year 2013 whereas 103, 149, 176 miRNAs were identified from the FD samples of Deh, Mun, Chit in the year 2014. On the other hand, a total of 166, 168, 154 and 174 miRNAs were identified from the GH samples of Deh, Mun, Chit, and Col-0, respectively (Supporting Information Table S4). The expression level of 170 commonly expressed miRNAs in each population from both the years was significantly correlated (Spearman rho correlation >0.8, P < 0.0001). Therefore, further analysis of miRNAs was carried out using only the 2013 data, because the global gene expression data of the same samples were available^53^ to predict and correlate the miRNA and mRNA expression.

### Highly overlapping expression of miRNAs in different populations

**Figure 2:**
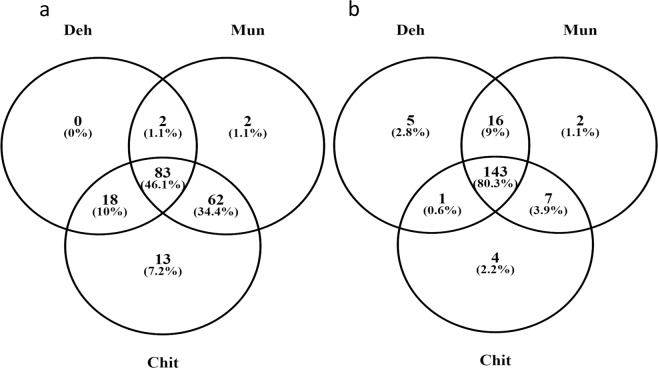
Venn diagram representing different commonly and uniquely expressed miRNAs in different populations under (**a**) field and (**b**) glass house condition.

**Figure 3:**
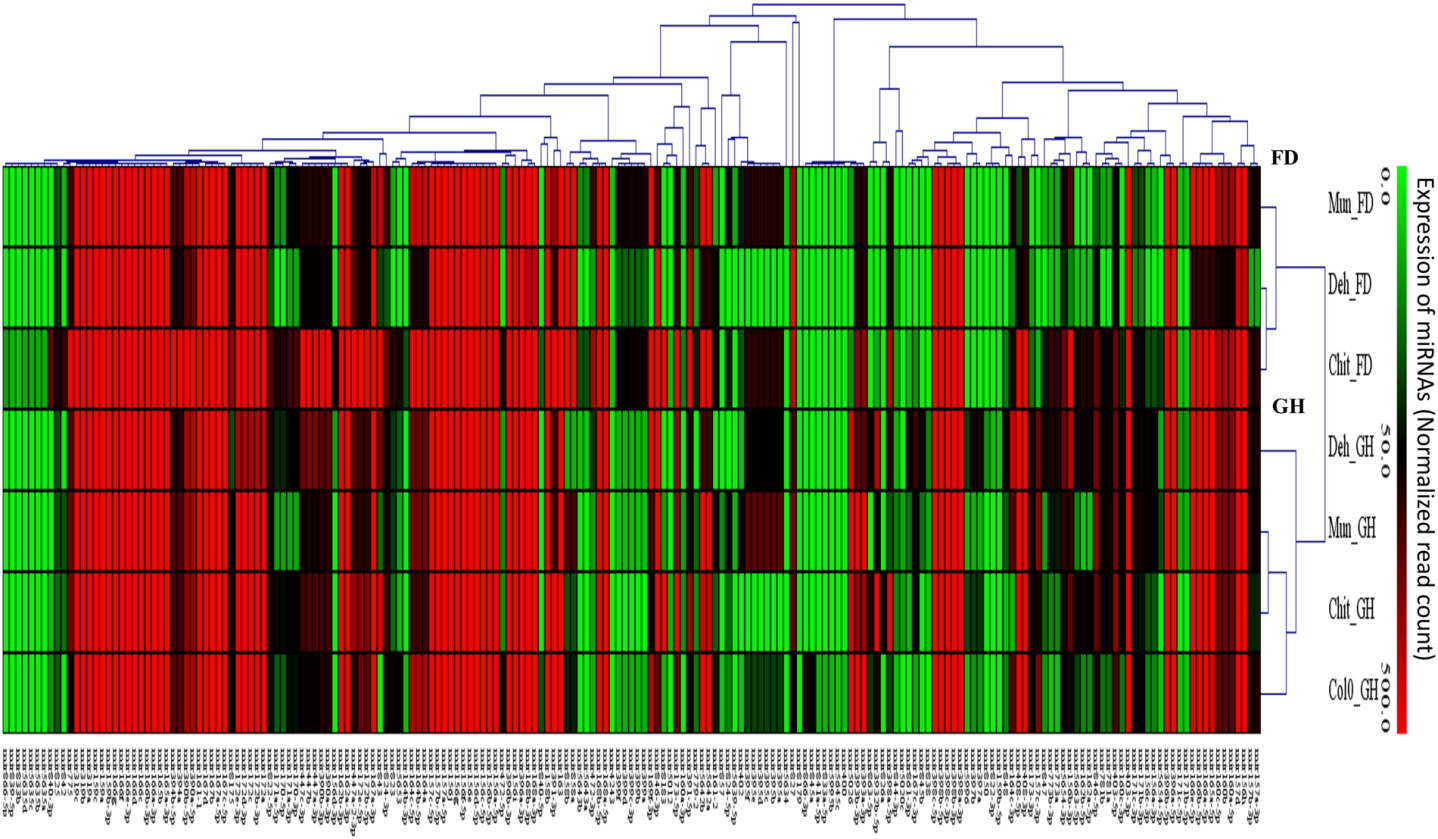
Hierarchical clustering of commonly expressed miRNAs in different populations displayed by average linkage clustering and Pearson correlation as the measurement of similarity.

Expression analysis of miRNA in FD samples of the three populations indicated that about 46.10% of miRNAs were commonly expressed in all the three populations. However, about 7.2% and 1.1% of total miRNAs expressed specifically in Chit and Mun, respectively but Deh did not have any population-specific expression of miRNA under field condition (Fig. 2a). In comparison to FD samples, the GH samples showed a higher number (80.3%) of commonly expressed miRNAs and, 2.8%, 1.1% and 2.2% of total miRNAs were specifically expressed in Deh, Mun, and Chit, respectively under glasshouse condition (Fig. 2b). miRNAs miR159b-3p and miR166a-3p were the two most highly expressed miRNAs in all the populations under both the conditions. Further to compare the miRNA diversity, we also calculated the Jaccard similarity index between the three populations under the two conditions (Supporting Information Table S5). We found that Deh and Chit have lowest Jaccard similarity index [Average: 0.36 (95% CI:0.36–0.37)], followed by Deh vs Mun [Average:0.38 (95% CI:0.32–0.37)] and Mun vs Chit [Average:0.40 (95% CI:0.42–0.47)] under FD condition. In case of GH, Deh vs Chit also showed lowest Jaccard similarity index of 0.39 (95% CI:0.38–0.42) followed by Chit vs Mun [Average:0.41 (95% CI:0.34–0.43)] and Deh vs Mun [Average:0.42 (95% CI:0.37–0.39)]. Further, hierarchical clustering of miRNAs (by their expression) from all the populations under both the conditions indicated that the clustering of the populations was specific to growth conditions rather than populations per se (Fig. 3).

### Differential expression of miRNAs in different populations under FD and GH conditions

**Figure 4:**
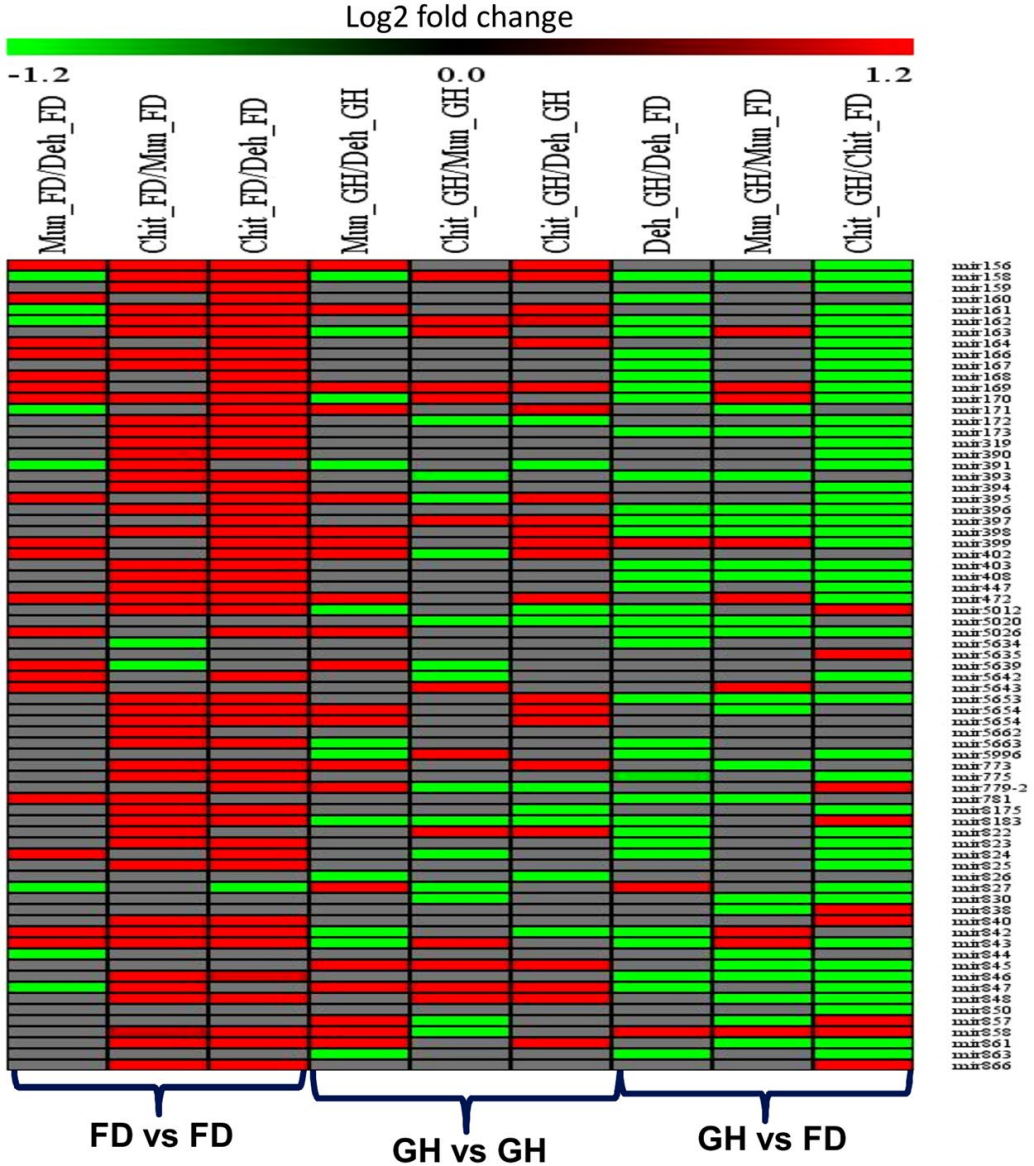
Heatmap of differentially expressed miRNAs between different populations under field and glasshouse conditions as well as between field and glasshouse condition for the same population. The color intensity from red to green indicates up and down-regulation, respectively.

We performed differential expression analysis of miRNAs between different FD samples as well as between GH samples taking the respective lower altitude population as the denominator for all the comparisons. A total of 99 (32 families), 133 (32 families) and 171 miRNAs (65 families) were differentially expressed between FD samples of Mun/Deh, Chit/Mun, and Chit/Deh, respectively. There was an increase in the number of differentially expressed miRNAs with the increase of the altitude of the native sites of the population. The miRNA families that were highly up-regulated in the middle altitude (Mun) populations as compared to the low altitude population among others were, miR169, (16 fold), miR395, miR402 (13 fold), miR5653, miR5026, miR781 and miR5639 (10 fold), etc. Similarly, the highly up-regulated miRNAs in the high altitude population (Chit) as compared to the low altitude population (Deh) among others were, miR169, miR8183 (16 fold), miR840, miR342, miR395, miR823, miR5653, miR781, miR847 (13fold), etc. (Fig. 4 and Supporting Information Table S6). Among these miRNAs, the expression level of 30 miRNAs was higher in both medium and the high altitude populations as compared to the low altitude population. We refer these miRNAs as high altitude associated miRNAs (detailed below). Moreover, there were 30 miRNAs whose expression increased with an increase in the altitude. None but one miRNA’s expression decreased with the increase in the altitude (Supporting Information Table S7).

To check the miRNA expression pattern in the absence of altitude-associated environmental factors, plants were grown under a common environment in GH condition. This may indicate population-specific expression under a common environment. The differential expression analysis of miRNAs between Mun/Deh, Chit/Mun, and Chit/Deh indicated that the number of differentially expressed miRNAs reduced up to 25–50% as compared to the FD. There were a total of 76 (38 families), 61 (30 families) and 83 (34 families) miRNAs which expressed differentially between Mun/Deh, Chit/Mun, and Chit/Deh, respectively. Some of the highly differentially expressed miRNAs between Mun/Deh were miR842 (12 fold), miR779, miR857, miR8183, miR5020, and miR5639 (11 fold), etc., between Chit/Mun were miR163 (13 fold), miR826 (12 fold), miR5012, miR840 (10 fold), miR163, miR158 (2 fold) etc. and between Chit/Deh were miR8183 (16 fold), miR826 (13 fold), miR842, miR779 (11 fold) and miR164, miR824, miR858, miR172 and miR156 (>2 fold) etc. There were a few miRNAs which were highly down-regulated in higher altitude populations as compared to the lower altitude population. For example, miR399 and miR395 were down-regulated more than nine-fold in Chit_GH as compared to Deh_GH. Also, there were a few miRNAs like miR156, miR157, miR164, and miR319 which were up-regulated in Chit and Mun under both the conditions as compared to Deh (Fig. 4 and Supporting Information Table S6).

### Differential expression of miRNAs between FD and GH

Comparison of miRNA expression level of the same population grown under field and controlled condition may indicate an environmental influence on miRNA expression pattern. The number of miRNAs and their fold change decreased in the GH samples as compared to the corresponding FD samples (taking FD samples as the denominator). A total of 92 (43 families), 79 (30 families) and 150 (44 families) miRNAs expressed differentially between GH and FD samples of Deh, Mun, and Chit, respectively. In Deh, 38 miRNAs were up-regulated, and eight miRNAs were down-regulated, whereas in Chit, 10 and 34 miRNAs were up and down-regulated in GH, respectively (Fig. 4 and Supporting Information Table S6). While in case of Mun, five miRNA were up-regulated and 23 miRNA were down-regulated. Interestingly, miR397 expressed in all the populations only under GH condition, while miR858 and miR398 had higher expressions in all the FD and GH samples, respectively. ANOVA test indicated that out of the 91 commonly expressed miRNA families between GH and FD, expression of 21 miRNAs was significantly affected either by population, their growing condition or by their interaction. There were four miRNAs (miR158, miR170, miR402, mir842) whose expression was governed only by its population. MiR395 was significantly affected by only growing conditions. Other miRNAs were either affected by both population and its interaction with growing condition (Supporting Information Table S8).

### High altitude associated miRNAs and their targets

We were further interested to find out differentially expressed miRNAs between high (Mun and Chit) and low altitude population (Deh). This may give us further insight on their role towards plants response under high altitude conditions. We did not find any miRNA which was down-regulated in the high altitude populations as compared to the low altitude population. However, there were 30 miRNA families that showed higher expression pattern in both the high altitude populations as compared to the low altitude population (Table 1). These miRNAs putatively target 271 different genes (Supporting Information Table S9). The expression level of these target genes were checked in the previously reported transcriptome data of the same samples^53^. A total of 168 target genes could be identified whose expression was negatively correlated with 26 miRNAs (Supporting Information Table S9). However, further analyses were carried out based on 30 miRNAs. This is because some miRNAs may regulate its target at the translational level which was not detected in this study. Out of these 30 miRNAs, nine are known to involve in developmental processes, while a few are known to govern salinity response (3), nutrients homeostasis (2), UV tolerance (3), secondary metabolites regulation (2) and cold response (2) etc. (Supporting Information Table S9).

**Table 1:** List of high altitude associated miRNAs and their relative expression in high altitude populations.

miRNA	log2 fold changes (Mun_FD/Deh_FD)	*P value*	log2 fold changes (Chit_FD/Deh_FD)	*P value*
miR169	16.90	2.264E-266	16.83	2.722E-254
miR395	13.48	1.3443E-25	13.54	1.20953E-26
miR402	12.09	3.3045E-10	11.54	3.35304E-07
miR5643	10.99	3.7933E-05	11.59	2.10334E-07
miR5026	10.77	0.00016275	10.94	5.66574E-05
miR781	10.60	0.0004297	12.71	2.65627E-15
miR823	10.51	0.00069823	13.43	1.28208E-24
miR843	3.46	4.672E-66	4.65	8.9869E-175
miR472	3.20	4.101E-25	4.58	5.84393E-79
miR824	2.75	2.1734E-30	2.96	2.11176E-37
miR161	2.22	0	4.17	0
miR5642	2.22	4.2932E-63	2.85	1.1435E-125
miR170	2.17	1.7757E-08	4.05	5.35272E-49
miR164	2.11	1.5051E-53	2.88	3.5535E-125
miR160	2.01	1.4446E-35	2.25	1.21419E-47
miR399	1.92	5.4883E-11	1.43	9.61914E-06
miR156	1.75	3.232E-206	3.09	0
miR168	1.68	0	2.65	0
miR171	1.40	7.9792E-07	3.09	2.92175E-50
miR166	1.27	0	2.62	0
miR165	1.25	0	2.61	0
miR163	0.98	7.894E-183	2.33	0
miR394	0.92	3.506E-06	2.38	1.10437E-57
miR390	0.87	8.4834E-16	2.28	3.8706E-158
miR162	0.65	7.1022E-91	3.20	0
miR319	0.45	1.765E-112	2.09	0
miR858	0.33	1.1586E-09	1.35	2.1153E-208
miR159	0.30	0	2.30	0
miR167	0.17	1.5401E-05	1.86	0
miR396	0.08	1.6216E-05	1.68	0

**Figure 5:**
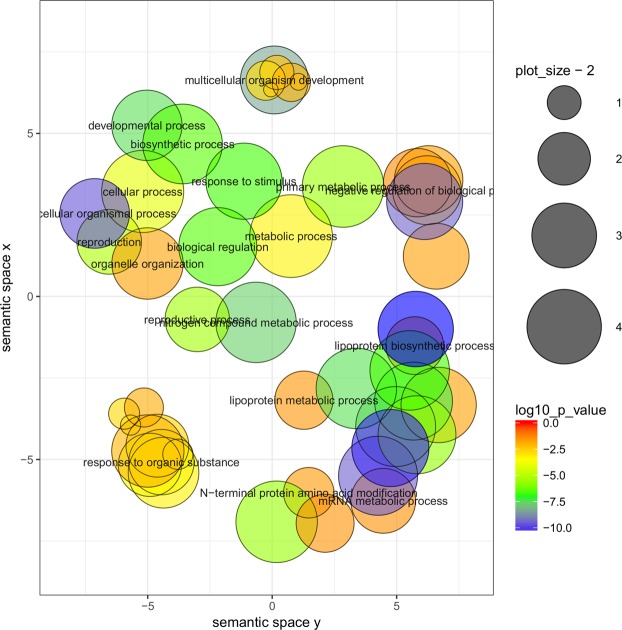
Gene ontology (GO) analysis of miRNA target genes. The predicted targets of miRNAs were analyzed using REViGO at FDR ≤ 0.05. The disc color gradient (Blue to red colors) indicates the degree of GO enrichment corresponding to log10 p-value, while the disc size is proportional to the frequency of the GO term in underlying GO annotation database. The spatial arrangement of discs approximately reflects a grouping of GO categories by semantic similarity. Discs representing similar GO terms are clustered closer together than discs representing unrelated GO terms.

To gain further insight into the functional role of these identified miRNA targets, the agriGO analysis was performed. The 168 target genes were enriched in 154 different GO terms (p < 0.05). The resulting GO terms were further summarized and clustered by REViGO. The over-represented GO terms were developmental processes, response to the stimulus, regulation of gene expression, and reproduction. In the developmental process, anatomical structure development, leaf development, system development and vegetative to the reproductive phase transition of meristem were the major ones (Fig. 5).

### Climatic variation and its relation to miRNA expression

The two principal components in PCA analysis were able to explain all of the variability of the climatic factors among the three sites (PC1- 76.38%; PC2- 23.62%) (Supporting Information Figure S3). The principal component scores calculated for these two significant PCs were correlated with the normalized miRNA expression values. A significant positive correlation of five miRNAs with the PC1 was observed, while none were significantly correlated with PC2. The PC1 was loaded negatively mostly with temperature related terms indicating a negative correlation between temperature and miRNA expression. Further, the correlation between commonly expressed miRNA and climatic conditions prevailing during the growing periods indicated 32 miRNA families were strongly correlated with radiation and temperature (R-values > threshold (0.98) and P-value > 0.05), of which 16 miRNAs were positively correlated with AR and 16 miRNAs were negatively correlated with AT. None of the miRNAs families were correlated with average precipitation during growing condition (Supporting Information Table S10).

### Identification of novel miRNAs

Twenty-nine novel miRNAs were identified from the pooled libraries of FD and GH using miRCat as described in materials and methods. The secondary structure, minimum folding energy and precursor sizes of these novel miRNAs are given in (Supporting Information Table S11 and Supplementary Figure S4). Out of 29 novel miRNAs, five originated from intronic, and 24 originated from the intergenic region of different genes. Twenty-one novel miRNAs expressed commonly in all the three populations under both the conditions. Twenty-two novel miRNAs expressed differentially between Chit-FD/Deh-FD while 21 novel miRNAs expressed differentially between Mun-FD/Deh-FD. Further, out of these 29 putative novel miRNAs, 20 miRNAs expressed in Col-0 but were never reported earlier. The remaining nine putative novel miRNAs were specific to the Himalayan populations. Among these nine miRNAs, five miRNAs expressed in all the populations whereas two miRNAs were expressed only in Chit and one was specific to Mun (Supporting Information Table S12).

### Validation of differentially expressed miRNAs by qRT-PCR

The expression level of miRNAs as determined by the NGS data was validated further using qRT-PCR. Sixteen miRNAs which showed differential expression in the field vs field comparison were selected for qRT-PCR analysis. Out of 16 miRNAs, 14 miRNAs exhibited similar expression pattern in qRT-PCR as was observed in NGS data (R^2^ = 0.41) (Supplementary Information Figure S5).

### Relative expression of different arms of miRNA hairpins in GH

Differential expression of different arms of hairpins is an indication of the differential stability of miRNA duplex. In general, “no arm annotated” type miRNAs were predominant in the Himalayan populations followed by “both arm annotated” (5p and 3p) miRNAs. “No arm annotation” miRNAs ranged from 51% (Deh and Mun) to 47% (Chit and Col-0). “Both arms annotated” miRNAs were found to be maximum in Col-0 (35.23%) followed by Deh (34.71%), Mun (33.60%) and Chit (33.62%), respectively. On the other hand “one arm 3p annotated” miRNAs were highly expressed in Chit (11.50%) followed by Col-0 (8.40%), Deh (8.20%) and Mun (8.19%) while “one arm 5p annotated” miRNAs were higher in Col-0 (8.40%) followed by Chit (7.0%), Mun (6.50%) and Deh (5.70%), respectively (Supplementary Information Figure S6a). Further, a total of 56 commonly expressed “both arms annotated” miRNAs were detected in the Himalayan populations as well as Col-0. The abundance ratio of 5p and 3p was estimated for these miRNAs. Out of these 56 miRNAs, 40 miRNAs exhibited significant differences in the expression of 5p and 3p arms (p < 0.05) in all the populations (Supplementary Information Figure S6b). The log2 fold ratio of 5p and 3p of some of the miRNAs in a particular population was higher than the others. For example, the log2 fold ratio of 5p and 3p of miR160a in Deh was 14 whereas it was three in both Mun and Chit. Similarly, miR164c showed a very contrasting expression pattern of both the arms in different populations. The log2 fold ratios of 5p and 3p of miR164c were 0.78, 0.37, and 3.5 in Deh, Mun, and Chit, respectively (Supplementary Information Figure S6c).

### Analysis of isomiR diversity

**Figure 6:**
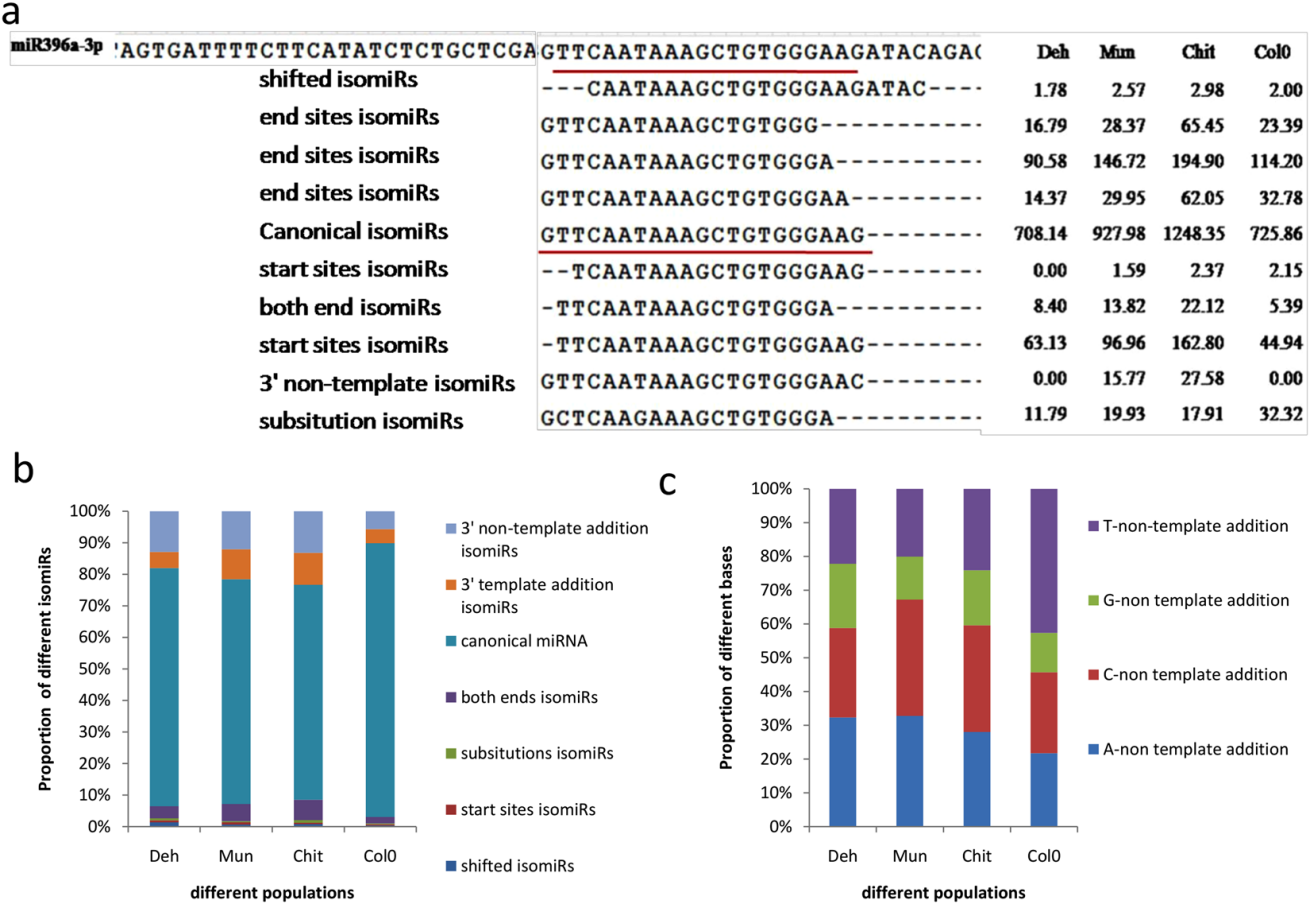
Schematic representation of different forms of isomiRs and their expression pattern in different populations including Col-0. (**a**) For an example, the sequence of different isomiRs of miR396a is shown; depending on the variation from annotated mature miRNA sequence (underlined in red) is shown. The values under each population indicates number of normalized read counts corresponding to the respective isomiRs forms, (**b**) Cumulative bar plots showing the proportion of different types of isomiRs for all identified miRNA, (**c**) Cumulative bar plots representing the proportion of different nucleotides added to the 3′ end of non-template addition type of isomiRs.

IsomiRs were classified into seven classes by the pattern of sequence variation from that of the canonical miRNA (Fig. 6a). As expected the canonical form was dominating in all the populations. Among the non-canonical isomiRs, the 3′ non-template addition (3′ NTA) forms were the dominating one (5–13%) followed by 3′ template addition (3′ TA, 4% to 10%). The representation of other non canonical isomiRs including start site isomiRs (0.69% to 0.34%), substitution isomiRs (0.85% to 0.31%) and both end isomiRs (6.45% to 2.09%) were very less.

At the population level, there were variations in the dominance of different isomiRs. For example, the abundance of 3′ TA form was 5% more in Chit and Mun as compared to Deh and Col-0. On the other hand, the expression of 5′ shifted isomiRs was the highest in Deh and the least in Col-0 (Fig. 6b). We further analyzed different bases (cytidine, uridine, adenosine, and guanosine) added in 3′ NTA isomiRs in different populations. It was observed that adenosine was the most dominating nucleotide added at 3′ end of isomiRs in Deh population, representing about 32% of the total fraction. In both Mun and Chit, cytosine was overrepresented (34% and 31%) followed by adenosine (32% and 28%). In Col-0, uracil addition (42%) was the most dominating one. Interestingly, we could not identify the addition of guanosine in any of the libraries (Fig. 6c).

**Figure 7:**
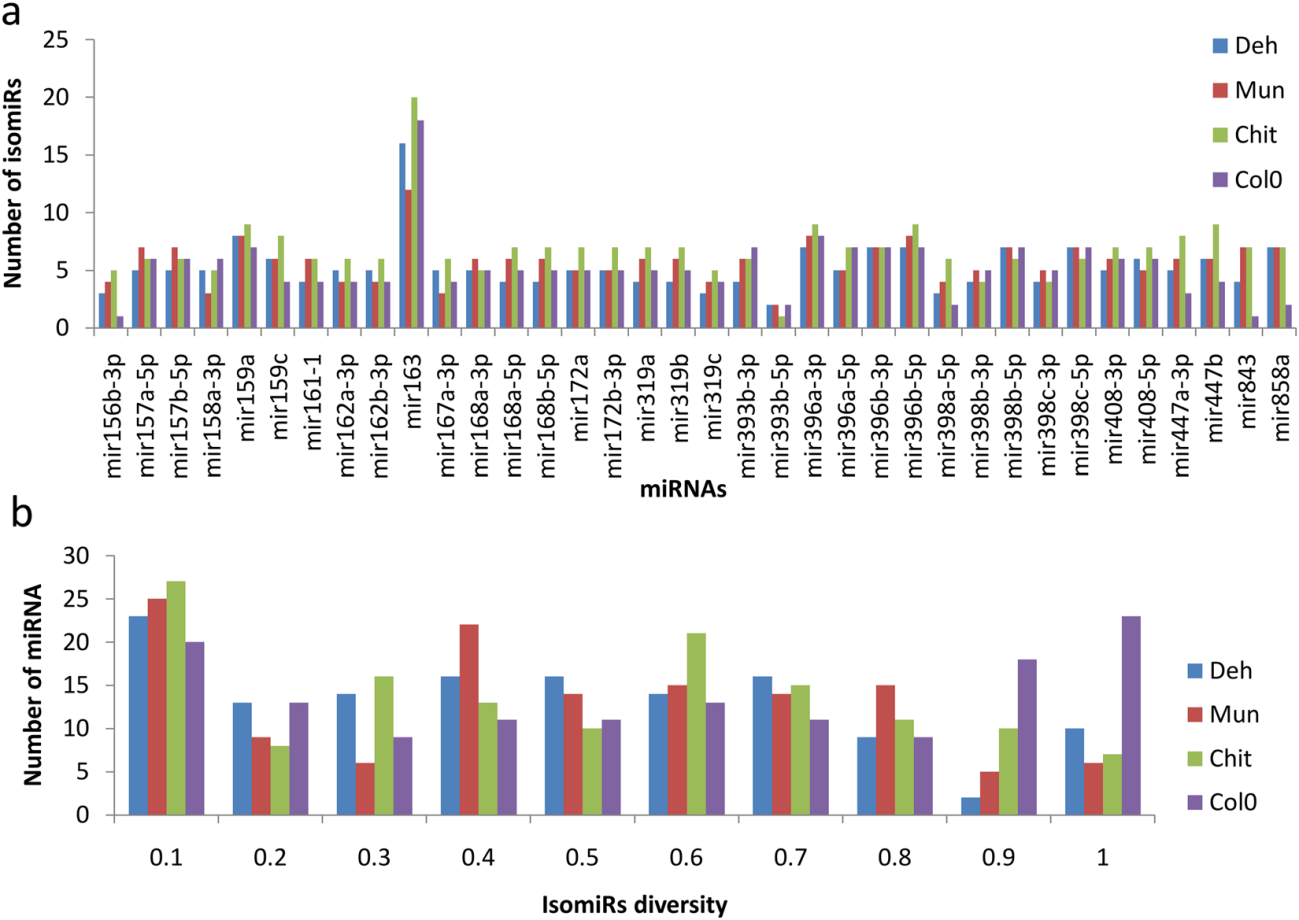
Different isomiRs of different miRNAs and their diversity in different populations. (**a**) Bar plots showing the number of different isomiRs identified for different miRNAs. A total of 36 miRNAs were found with the different number of isomiRs in different populations for a particular miRNA, (**b**) Barplot showing the distribution of isomiR diversity in the four populations.

At the individual miRNA level, miR163 exhibited maximum isomiRs in Chit (19), followed by Col-0 (17) Deh (15) and Mun (11) (Fig. 7a). Further, the mean miRNA diversity was estimated by quantifying the expression of dominant isomiRs over the total expression of that miRNA. A total of 23, 25, 27 and 20 miRNAs exhibited the highest diversity in Deh, Mun, Chit, and Col-0, respectively whereas there were 10, 6, 7 and 23 miRNAs with the lowest diversity in Deh, Mun, Chit, and Col-0, respectively (Fig. 7b).

### The differential phenotypic response of high and low altitude populations

**Figure 8:**
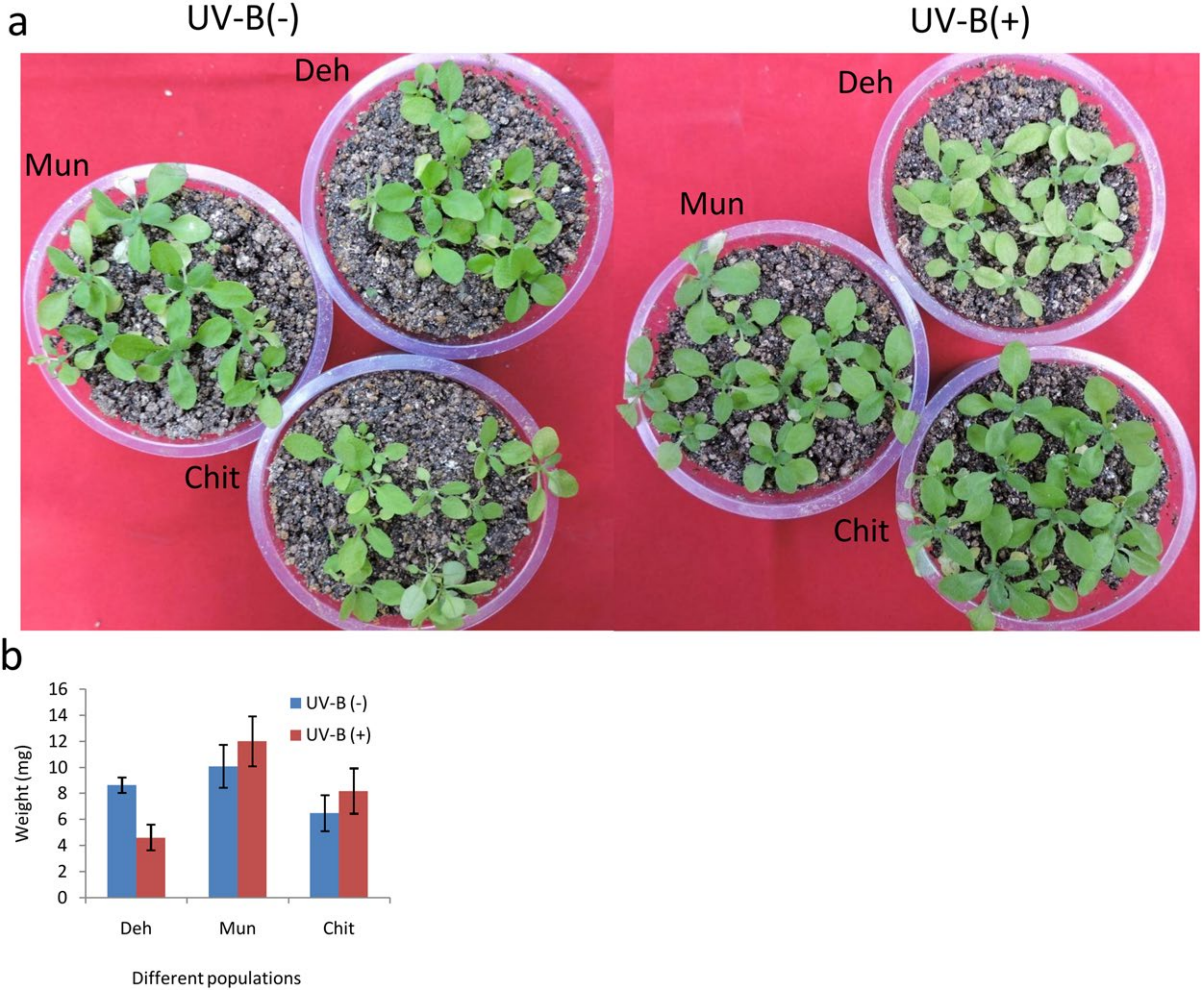
Effect of UV-B radiation on different populations. (**a**) Image of twenty-eight days old seedlings grown under UV-B treatment for 7 days. (**b**) Weight gain or loss of seedlings was calculated from the dry mass of the seedlings after 28 days.

Among other climatic factors, UV radiation is one of the dominating factors in high altitude area. Our results suggested that there was a higher expression of UV responsive miRNAs in high altitude populations as compared to the low altitude population. To test if the high altitude populations were more adaptive in UV condition as compared to the low altitude, the populations were grown under UV light. The UV- B treatment affected the leaf number, leaf viability (tested as the number of dead or yellow leaves), leaf width, leaf area, petiole length and rosette area in all the populations (Supporting Information Table 13). However, low altitude population, Deh exhibited the maximum number of yellow/dead leaf as compared to the other two high altitude populations (Fig. 8a). Similarly, the gain in dry weight of the high altitude populations was more than the low altitude population when compared with their respective control (Fig. 8b).

### Differential lignin biosynthesis under GH and FD conditions may be regulated by miR397 and miR858

**Figure 9:**
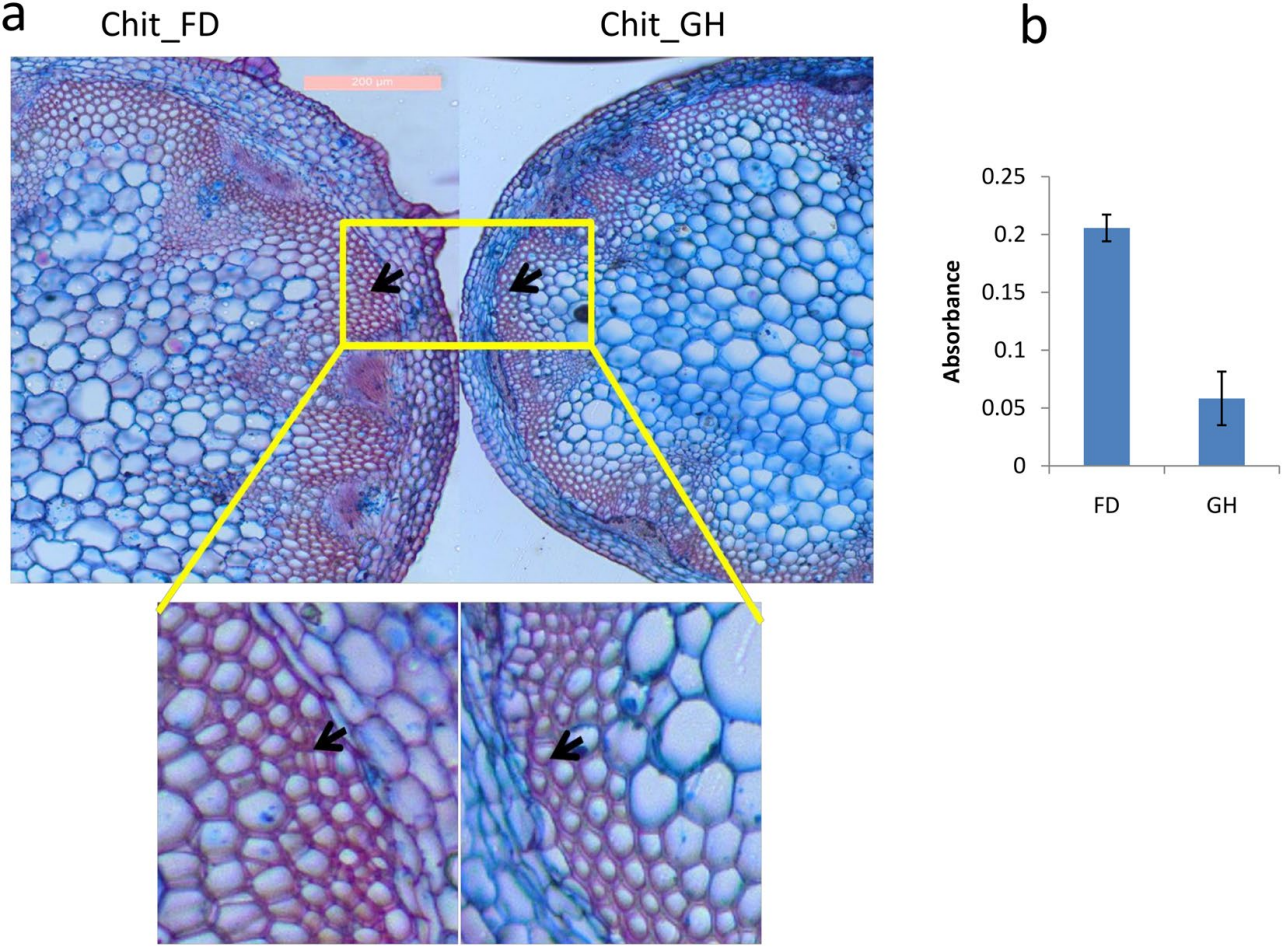
Lignin quantification of field and glasshouse grown plant. Lignin deposition was visualized under the light microscope using safranin staining after cross-sectioning of the stem. (**a**) Dense color indicates higher lignin (arrow) in FD samples and lighter color indicates lower lignin deposition in GH samples. (**b**) Absorbance measured at 280 nm for indication of relative lignin content between FD and GH.

It was observed that there were significant differences in the expression of miR397 and miR858 between GH and FD samples of the same population. To further validate its expression pattern between GH and FD samples, we grew the plants of the three populations in the pot filled with soilrite under common field condition. qRT-PCR analysis of miR397 and miR858 of these samples and GH samples also indicated that expression of miR397 was much less in common field condition than the GH condition whereas expression of miR858 was more under FD condition than the GH condition for all the three populations (Supplementary Information Figure S7). Since miR397 and miR858 are known to regulate lignin biosynthesis^36,61^, we visualized and estimated lignin content in the stems of these samples. As expected, the results indicated that lignin was higher in FD samples (absorbance 0.2) as compared to the GH plants (absorbance 0.05) (Fig. 9 and Supplementary Information Figure S8).

## Discussion

The populations of *A*. *thaliana* growing along the altitudinal range of the West Himalaya inhabit a wide climatic condition ranging from sub-tropical to temperate^51,52^. Among these populations, Chit represents one of the highest elevation habitat reported so far for *A*. *thaliana*. The miRNA expression analysis of these populations led us to identify a total of 252 known and 29 novel miRNAs. We observed a contrasting difference in the read length distribution of sRNA between field and controlled grown plants. The 21-nt sized sRNAs were dominated in the FD samples and the 24-nt sized small RNAs were more in GH samplesThe differential length distribution might be due to activities of different types of DCL enzymes^62^ and/or by over-representation of different miRNA variants and their isomiRs^41^ expressed under different environmental conditions. Small RNA of 24-nt sized are mostly derived from the DCL3 activity. They largely control chromatin level modifications and DNA methylation through the RdDM pathway^63^. It remains to be seen if the RdDM pathway is more active under GH condition than FD condition. Further, miRNA expression level and its association with climatic factors (23 bio-climatic factors) indicated only five miRNAs those were negatively influenced by annual temperature. However, when the climatic factors prevailed during the growing period only were considered, 32 miRNAs exhibited an association with average temperature and radiation. This was exemplified by the fact that in clustering analysis, the commonly expressed miRNAs showed condition specific grouping rather than their population affiliation. It was interesting to note that some of the miRNAs expressed at increasing level with an increase in the altitude of the origin of the populations. These miRNAs might be tightly regulated by the environmental factors such as temperature and radiation etc, associated with the altitudinal gradient. Further, the number as well the expression level of most the miRNAs decreased under glasshouse condition as compared to the field samples. This suggested that plants under a benign condition require less regulatory mechanisms than that of harsh field conditions. Interestingly none of the miRNA expression was found to be influenced by precipitation.

The high altitude populations are exposed to harsh environmental conditions as compared to the lower altitude population. Thus, they may show differential expression of stress-related miRNAs. We identified 30 miRNAs those were differentially expressed between high (Chit and Mun) and low (Deh) altitude populations. A few of these miRNAs are involved in regulating different abiotic stresses including drought, UV, cold, etc. For example, expression of miR169 was 16 fold higher in high altitude population than the lower one and miR169 has been shown to respond to various environmental signals including low temperature^64^, drought^65^, light^66^, and salinity^67^. Accordingly, its target NFYA5 (a nuclear factor Y, sub unit A) involved in regulating flowering in *A*. *thaliana* were down-regulated in higher altitude populations^68^. This might promote early flowering to complete the life cycle before the onset of early snowfall in the high altitude area. Similarly, miR395, involved in regulating sulfate assimilation^69^ was significantly higher (13 fold) in high altitude population. Though we do not have empirical data on the sulfur content of the soils, it is reported that in general sulfur content of high altitude area is less as compared to the lower one^70,71^. The miR402 expressed at more than 12 fold higher in high altitude populations. This miRNA is known to regulate seed germination and growth of seedlings under salt stress by regulating DEMETER-LIKE protein3^72^. Accordingly, the expression of DEMETER-LIKE protein 3 was also low in higher altitude populations. Higher expression of these and other miRNAs indicate that the high altitude populations have evolved strategies to counter different abiotic stresses as opposed to the low altitude populations where such abiotic stresses were not dominant.

Among other abiotic stresses, light intensity including UV radiation was particularly higher at high altitude region as compared to the low altitude^53^. UV radiation is also known to increase by 10 to 12% with every 1000 meter increase in the altitude^73^. As expected, the expression of UV responsive miRNAs like miR156, miR160, miR167, and miR170 was higher at high altitude populations as compared to the low altitude population. These miRNAs target different SPLs, ARFs, scarecrow-like transcription factor, and control different development process^7,28^. The targets of these miRNAs were also found to be down-regulated in the high altitude populations. The overall growth, particularly leaf width and leaf number of this high altitude population was reported to be less as compared to the low altitude population^51^. This may be one of the strategies to minimize the UV and /or high light intensity absorption by the plant. This was further corroborated by the *in-vitro* exposure of these plants under UV-B. The high altitude population was more tolerant towards UV-B treatment as compared to the low altitude population. Beside leaf size and shape, leaf architecture is also influenced by environmental factors^74,75^. miR164 is known to regulate the serration of leaves by targeting NAC transcription factors in *Arabidopsis*^76,77^. miR164 was found to be significantly up-regulated in high altitude populations. Inconsistent with the observation, the high altitude populations had low serration as compared to the low altitude plant^51^ (Supporting Information Figure S9). Leaves with less serration exhibit lower photosynthesis, higher transpiration rate, and lower surface area and thus may act as a compensating mechanism with high radiation^78,79^.

**Figure 10:**
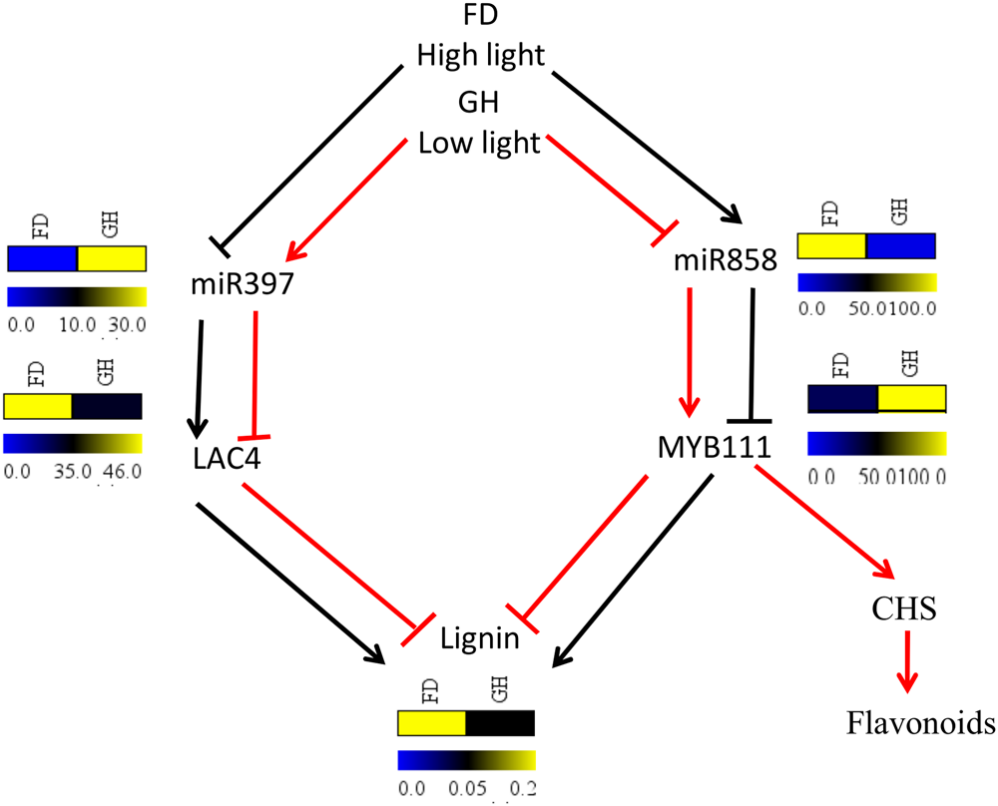
Schematic representation of the proposed hypothesis on lignin biosynthesis regulated by miRNAs. The red and black lines indicate the pathways operated in the glasshouse (low light) and field (high light), respectively. The heatmap for miRNAs and their targets was plotted against the normalized reads and for lignin, the heatmap was plotted against absorbance at 280 nm.

Secondary metabolites play important roles in plant growth and development particularly, under harsh environmental conditions^80^. miR858 is involved in regulating flavonoid biosynthesis pathway by targeting different types of MYBs^81^. The expression of miR858 is reported to be influenced by light^36^. However, our data suggest the expression of miR858 may also be influenced by the intensity of light. This is because there was an increase in the expression of miR858 with the increase in the altitude along with the concomitant increase in light intensity. Moreover, its expression in controlled condition (where light intensity was much lower than field) was much lower as compared to the field plants. But contrary to the earlier report^36^, we observed high altitude population (expressing the high level of miR858) was less vigor than lower altitude population^51^. It is to be noted here that earlier observation was reported on the basis of controlled grown plants. This might eliminate the impact of multiple environmental factors on the growth of plants. miR858 is also known to positively regulate lignin biosynthesis by targeting MYB111 transcription factor whereas miR397 negatively regulate lignin biosynthesis in plants by targeting laccase family of genes^36,61^. The expression of miR397 was much higher in all the population under GH condition than FD condition. As expected the lignin content was higher in FD plants than in GH plants as demonstrated by both quantification and microscopic observation. The high lignin content in plants under field condition provide mechanical strength to withstand damage caused by wind etc. whereas plants under GH condition are exposed to more benign condition and hence may suppress synthesis of the higher amount of lignin. Thus, we hypothesize that under high light intensity expression of miR858 is up-regulated which targets MYB111. It is reported that in the absence of MYB111 the flux of flavonoid biosynthesis is diverted towards lignin biosynthesis pathway^36,81^ leading to enhance lignin biosynthesis. On the other hand, high light intensity down regulates miR397 leading to more lignin biosynthesis by LAC4. Under low light intensity (glasshouse condition) reverse mechanisms may lead to lower lignin biosynthesis (Fig. 10). However, further experiments are needed to substantiate this hypothesis.

Among the differentially expressed miRNAs between low and high altitude populations, there were a few miRNAs which were expressed only in higher altitude populations. Similarly, a few novel miRNAs were present only in Indian populations of *A*. *thaliana*. It suggests these populations might have experienced a different selection pressure under different climatic condition leading to the development of new regulatory miRNAs. However, the functional aspects of both these groups of miRNA remain to be elucidated.

Besides the differential expression of miRNAs between high and low altitude population, there were significant differences in the abundance of 5p and 3p arms of some miRNAs in low and high altitude populations. Earlier reports indicated that selection of 5p and 3p arms of miRNA depends upon temporal, spatial and physiological condition^39,41^. It has also been suggested that since the 5p and 3p forms have different sequences, it might help plants to target different genes at the same time^42^. Over-representation of specific forms in different populations might help in the selection of more thermodynamically stable strands under a particular environmental condition. This may provide the fitness advantage under prevailing environmental conditions. Similarly, diversity of isomiRs has been shown to increase the efficiency of miRNA mediated target degradation and thermodynamic stability of target binding^82^. In animals, expression of isomiRs has been reported to be population and gender-dependent^83^. Our analysis of isomiRs indicated that Himalayan populations have more isomiR expression than Col-0. Moreover, the number and expression of isomiRs varied in all the populations, Chit having the highest percentage of isomiRs fraction than others. The increased expression of isomiRs in high altitude plants might help in thermodynamic stability of canonical miRNA and efficiency of target regulation. It is worthy to mention here that although there could be the possibility of occurrence of a few truncated miRNAs that might be the degraded product of primary or pre-miRNAs transcripts, but we followed stringent criteria of isomiR selection that excluded this possibility. Overall these results suggested that isomiR richness varied within the Himalayan populations as well as with respect to Col-0 suggesting a probable diverse role of isomiRs in plant adaptation across different climatic conditions.

## Conclusion

Populations exposed to different environmental conditions deploy different strategies to adapt to such conditions. As a part of such strategies, a large number of miRNAs were found to be differentially expressed between the low and high altitude populations of *A*. *thaliana*. These miRNAs were mostly influenced by the average temperature of the growing period of *A*. *thaliana*. Most of the high altitude associated miRNAs were found to be development and abiotic stress-related including salt, cold, temperature, UV and lignin biosynthesis. MiR397 and miR858 are involved in lignin biosynthesis and might be regulated by light intensity. The presence of higher isomiRs diversity in high altitude plants might provide an enhanced targeting of the genes that could lead to better adaptation of these populations. Several novel miRNAs including Himalayan population specific novel miRNA were identified. A more detailed study on these miRNAs and that of the high altitude associated miRNAs could provide further insight into the role of the miRNAs in the adaptation of these populations under such harsh environmental conditions.

## Supplementary information

Supplementary Figures

Supporting Information Table S1

Supporting Information Table S2

Supporting Information Table S3

Supporting Information Table S4

Supporting Information Table S5

Supporting Information Table S6

Supporting Information Table S7

Supporting Information Table S8

Supporting Information Table S9

Supporting Information Table S10.

Supporting Information Table S11

Supporting Information Table S12

Supporting Information Table S13
