# Supplementary material for: Modulation of miRNA expression in natural populations of A. thaliana along a wide altitudinal gradient of Indian Himalayas
Source: Sci Rep. 2019 Jan 24;9:441. doi: 10.1038/s41598-018-37465-y (PMC6345966; doi:10.1038/s41598-018-37465-y)
Supplement: Supplementary file 1 — Supplementary Figures [file 41598_2018_37465_MOESM1_ESM.pdf]

Modulation of miRNA expression in natural populations of *A. thaliana* along a wide altitudinal gradient of Indian Himalayas

Abhinandan Mani Tripathi[1, 2], Akanksha Singh[1, 2], Rajneesh Singh[1], Ashwani Kumar Verma[1], Sribash Roy*[1, 2]

1. Division of Genetics and Molecular Biology, CSIR-National Botanical Research Institute, Rana Pratap Marg, Lucknow, Uttar Pradesh, India
2. Academy of Scientific and Innovative Research (AcSIR), Anusandhan Bhawan, 2 Rafi Marg, New Delhi, India

* To whom correspondence should be addressed, E-mail: sribashroy@nbri.res.in

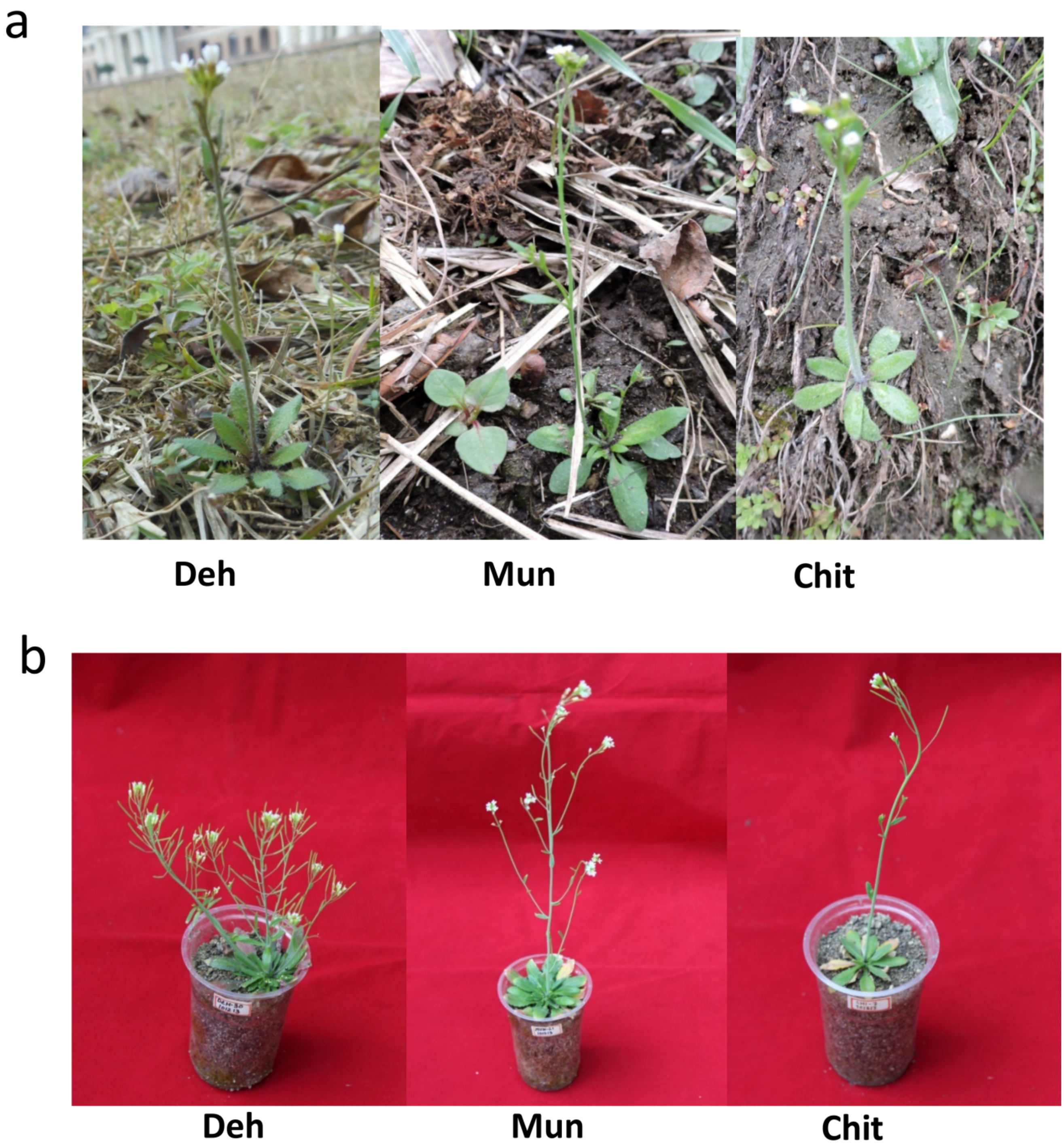

Supporting Information Figure S1. *Arabidopsis* plant of different populations growing in (a) Natural field condition (b) Glass-house condition.

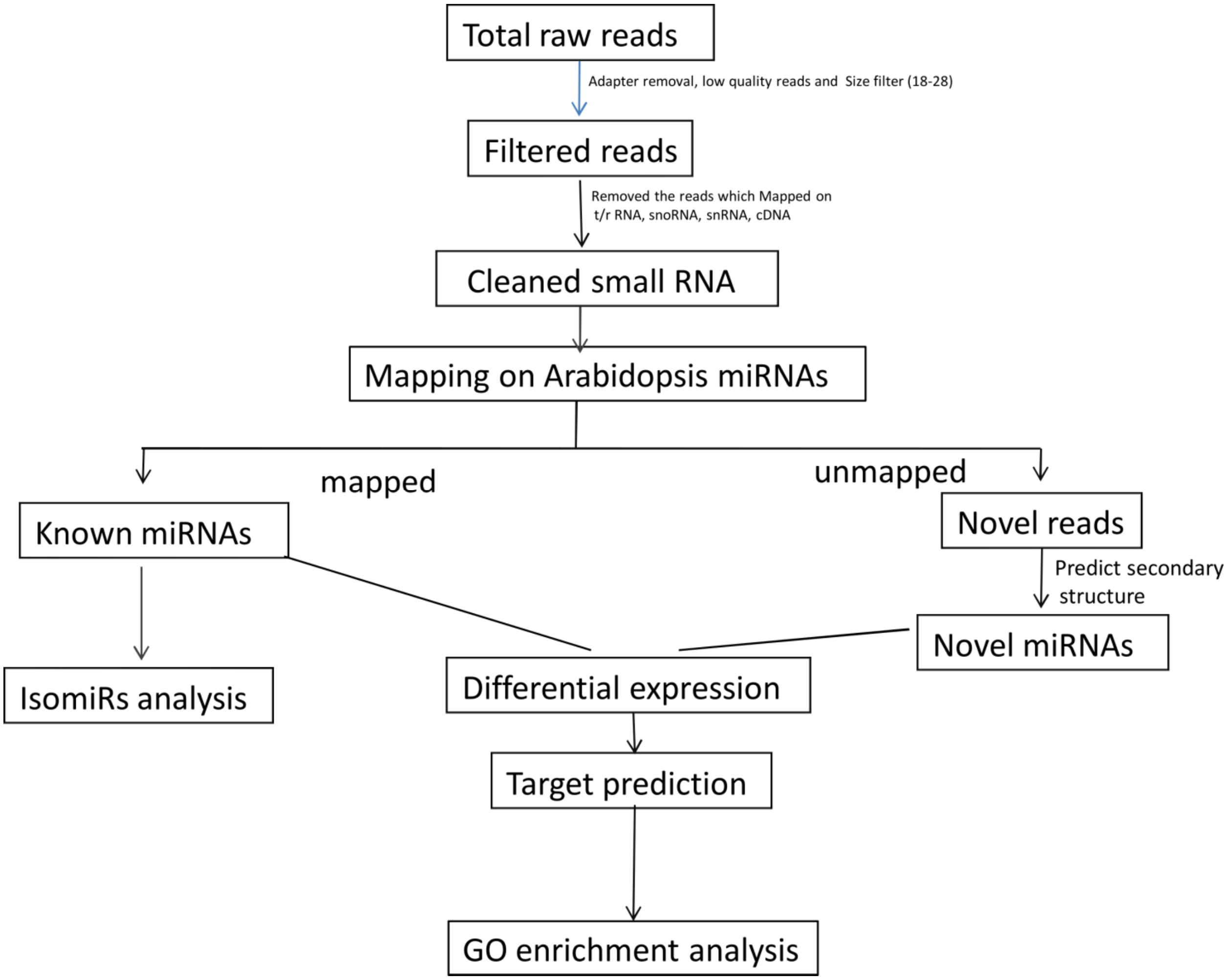

Supporting Information Figure S2. Schematic representation of small RNA analysis pipeline.

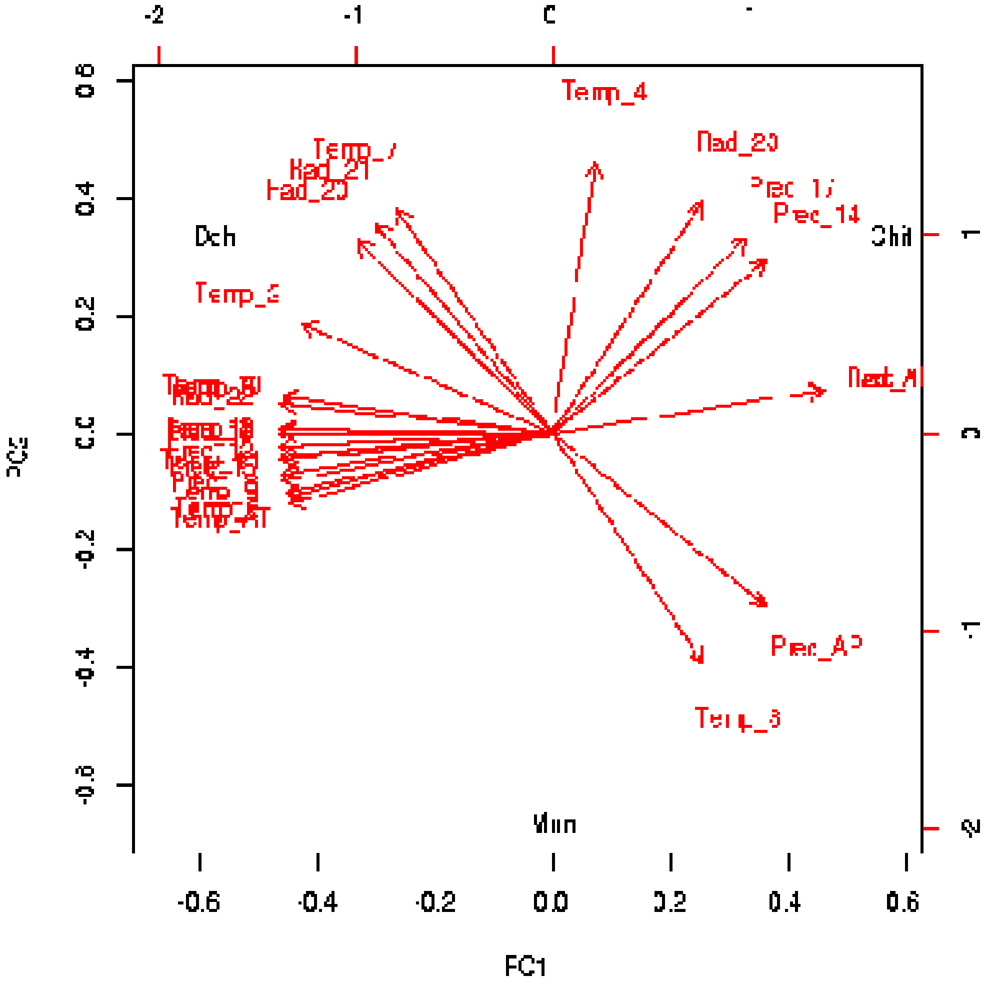

Supporting Information Figure S3. Principle component analysis (PCA) of different bioclimatic variables. The figure shows climatic differences among the sampling locations.

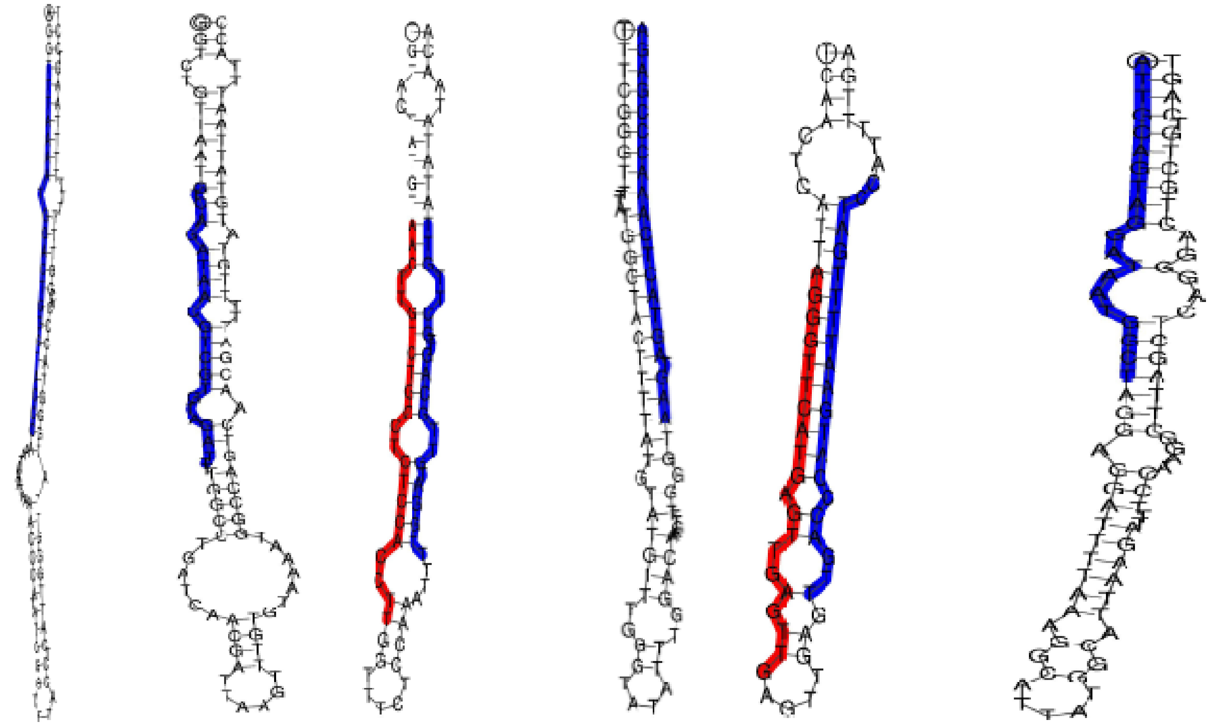

Noevl 1 Novel 2 Novel 3 Novel 4 Novel 5 Novel 6

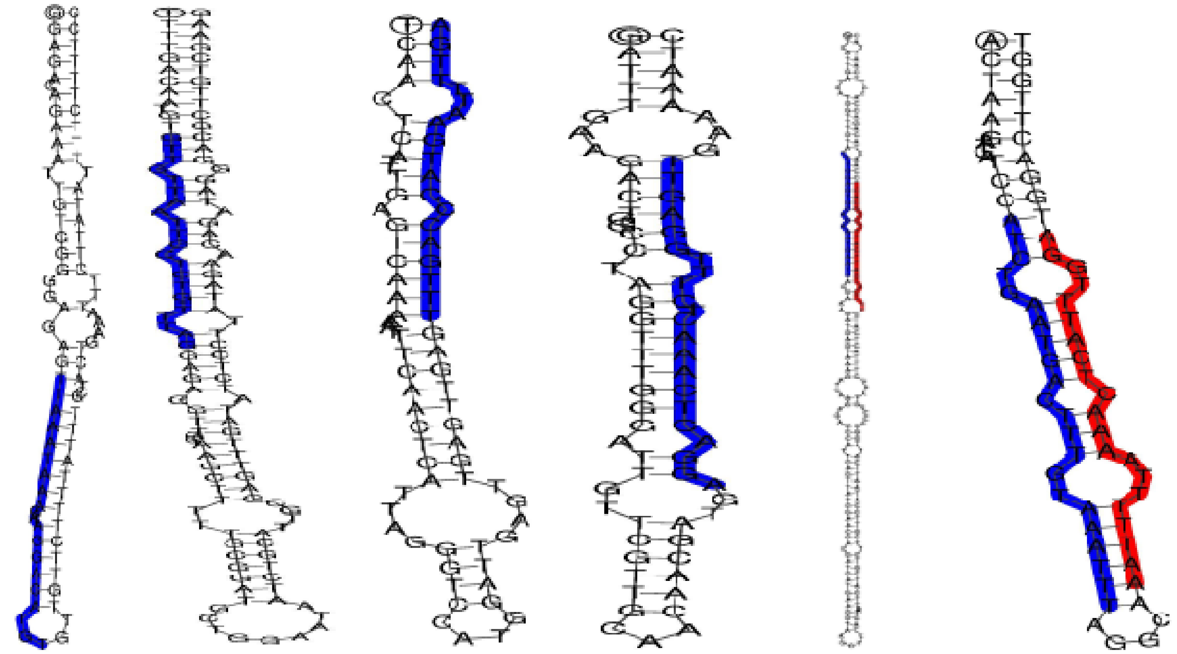

Noevl 7 Novel 8 Novel 9 Novel 10 Novel 11 Novel 12

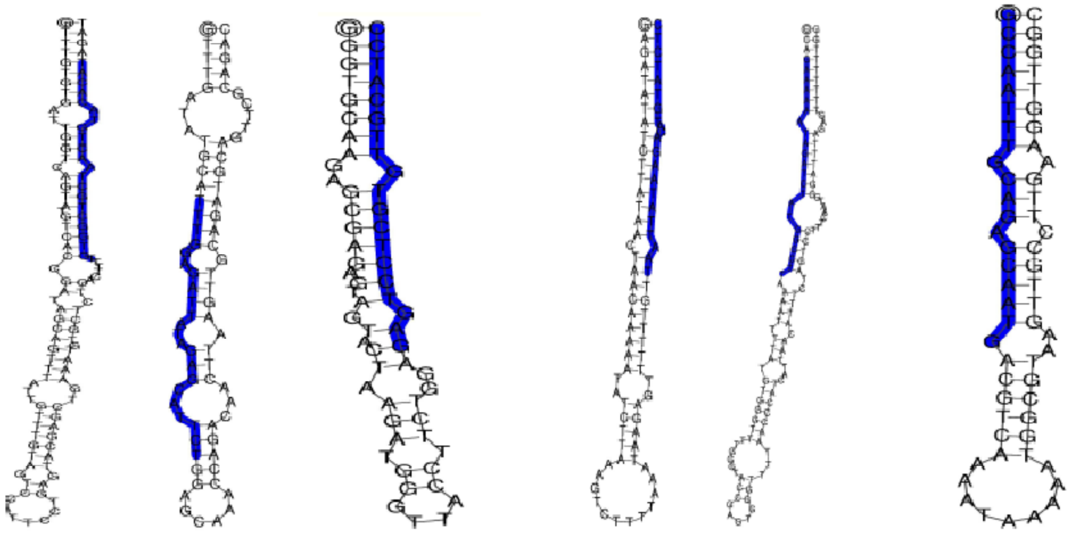

Noevl 13 Novel 14 Novel 15 Novel 16 Novel 17 Novel 18

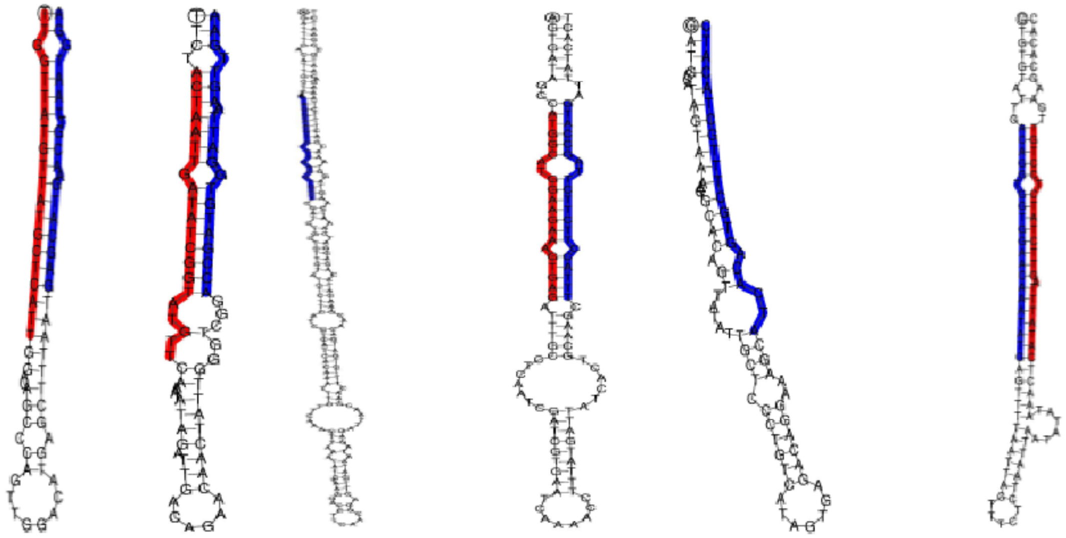

Noevl 19 Novel 20 Novel 21 Novel 22 Novel 23 Novel 24

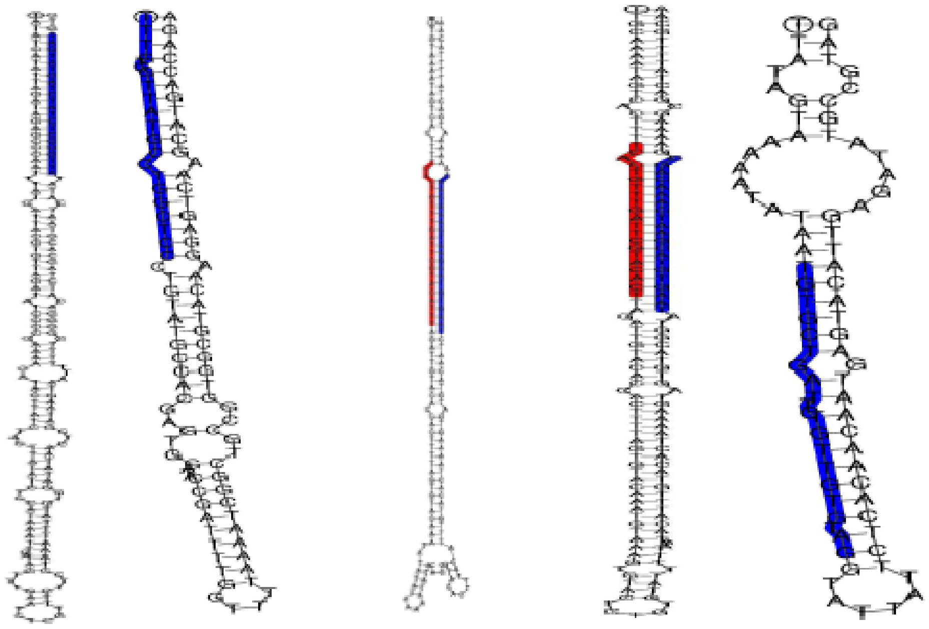

Noevl 25 Novel 26 Novel 27 Novel 28 Novel 29

Supporting Information Figure S4. Secondary structure of novel miRNA identified in this study. Blue and red color on stem indicated the position miRNA/miRNA*.

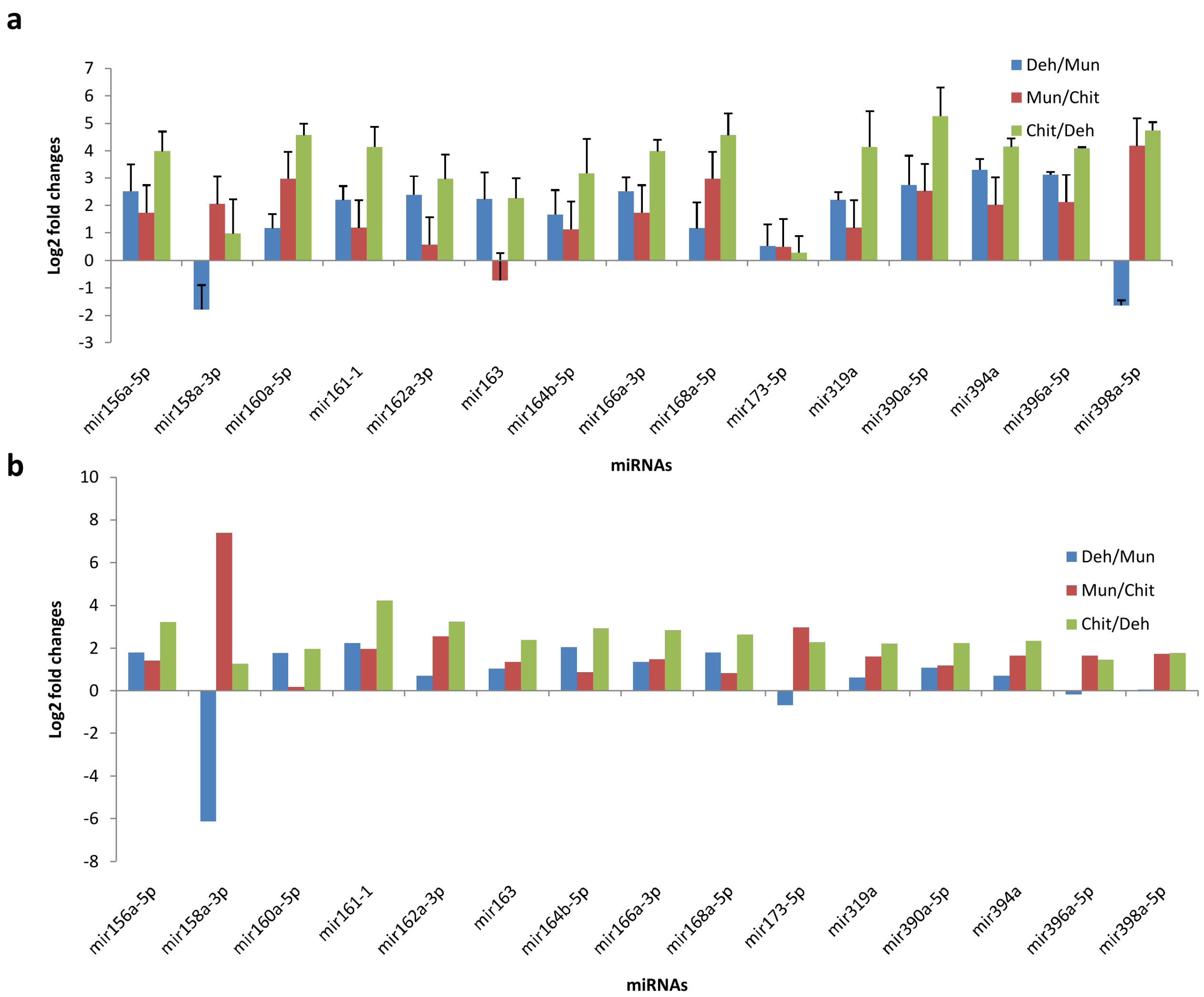

Supporting Information Figure S5. Validation of expression pattern of differentially expressed miRNAs by stem loop qRT-PCR. Expression pattern of randomly selected sixteen differentially expressed miRNAs between different population. (a) miRNA expression qunatified by using stem-loop qRT-PCR, (b) miRNA expression measured from NGS data.

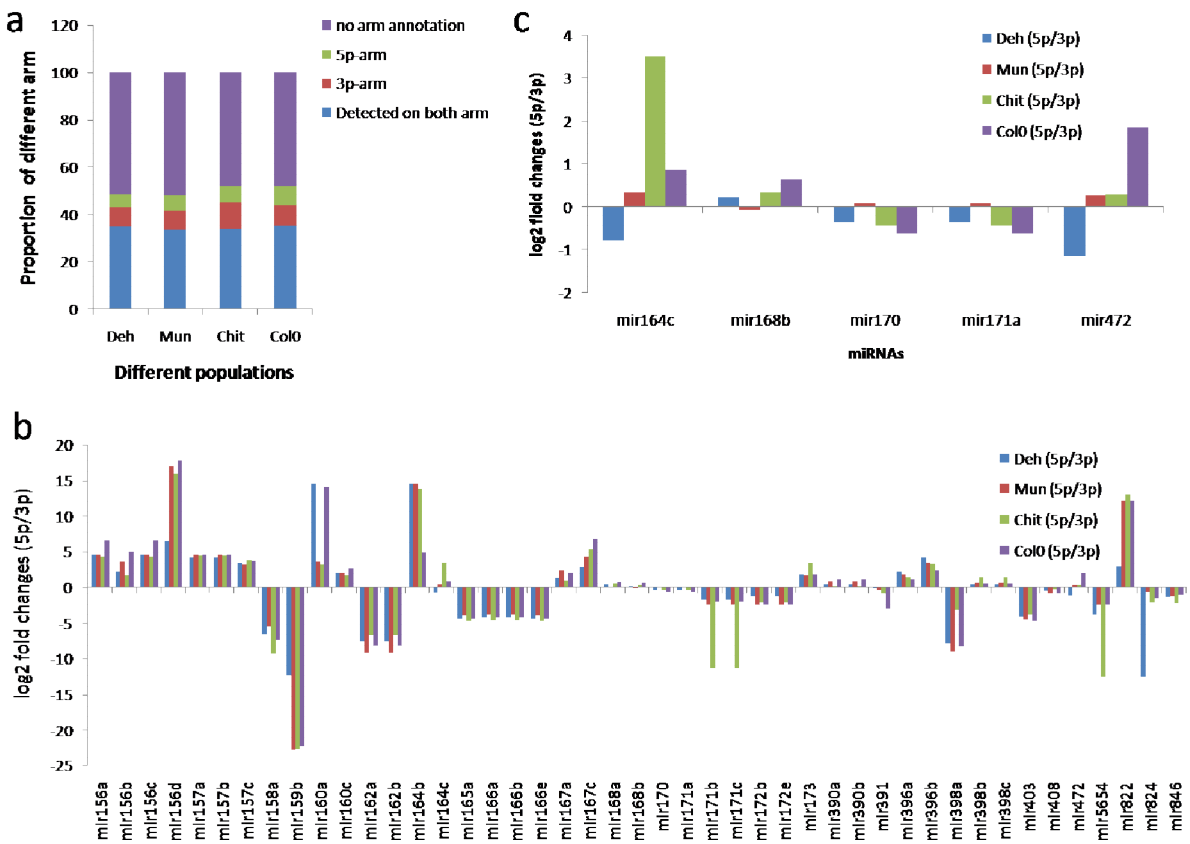

Supporting Information Figure S6. Population wise changes in the arm expression of different miRNAs. (a) Proportion of different arm of miRNA in different populations, (b) 5p/3p ratio of different arms in different populations (c) 5p/3p ratio of some miRNAs which showed contrasting difference in different populations.

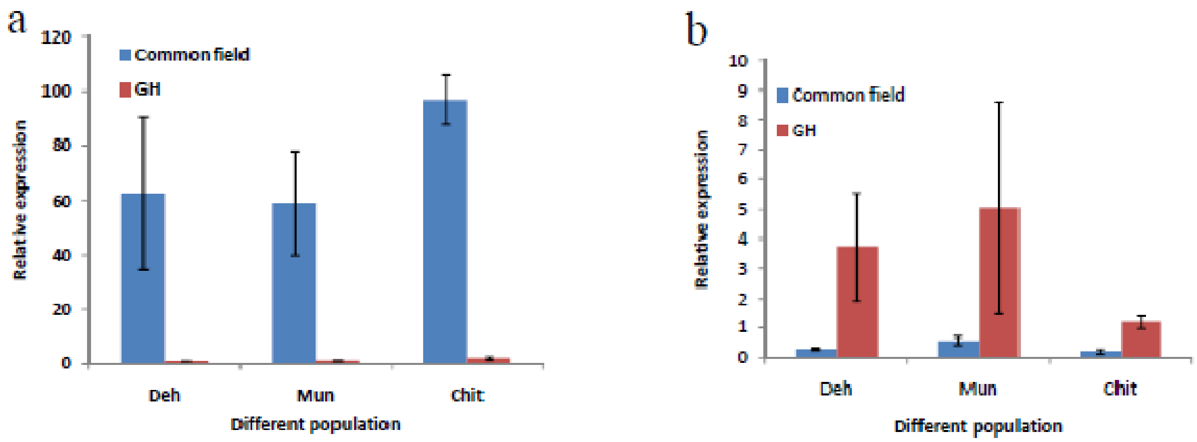

Supporting Information Figure S7. (a) Differential expression of miR858 and (b) miR397. The differential expression performed of the plant growing in common field condition by using stem loop qRT-PCR.

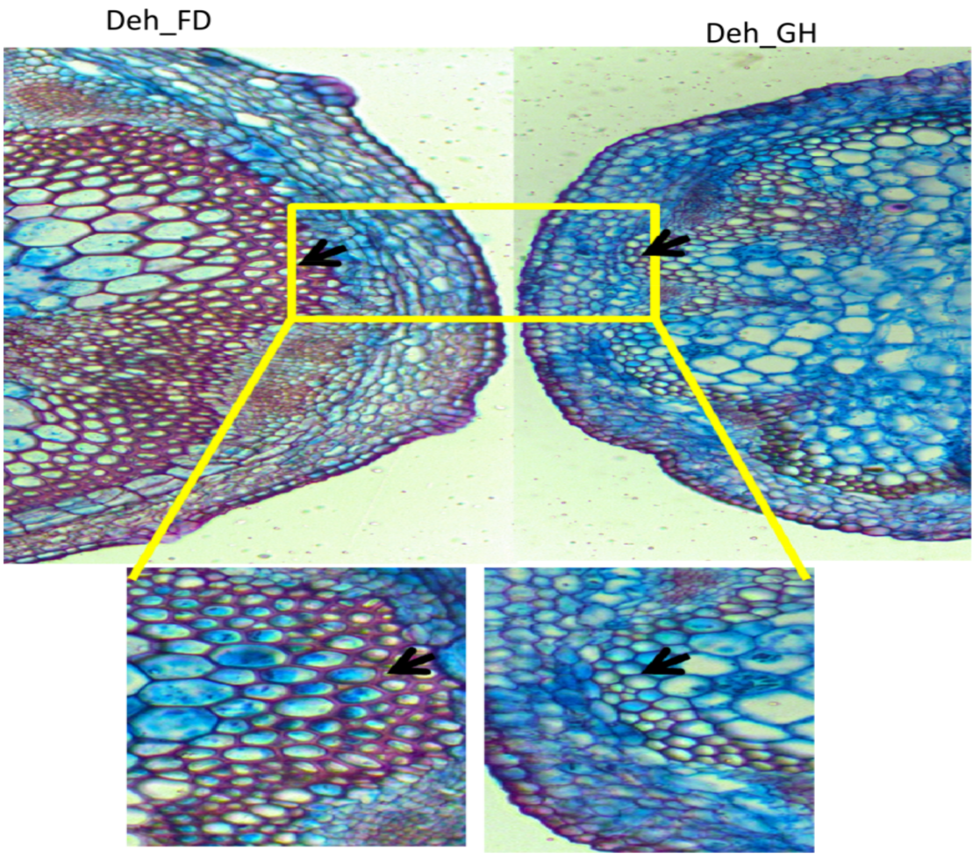

Supporting Information Figure S8. **Lignin quantification of field and glasshouse grown plant.** Lignin deposition was visualized under light microscope using safranin staining after cross-sectioning of stem. Dense color indicates higher lignin (indicated by arrow) in FD samples and lighter color indicates lower lignin deposition in GH samples.

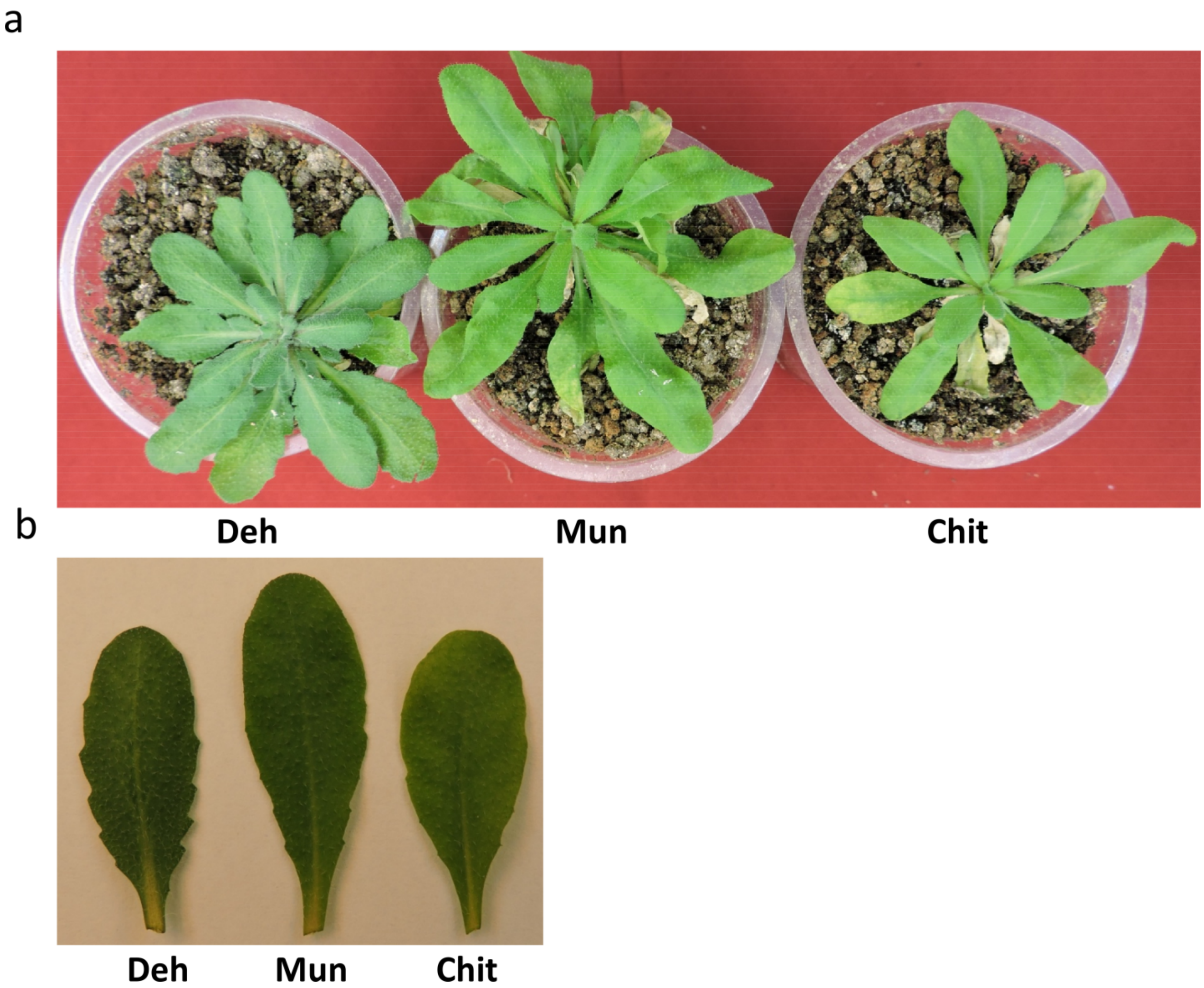

Supporting Information Figure S9. Photograph indicating the contrasting leaf serration in different populations.

Supporting Information Legends

Supporting Information Table S1. List of primers sequences that were used in this study.

Supporting Information Table S2. Interpolated bioclimatic data: 26 bio-climatic variables derived from monthly mean data of the four population sites. The table includes three seasonal variables derived for this study (highlighted in yellow).

Supporting Information Table S3. Categorization and abundance different small RNAs from different libraries.

Supporting Information Table S4. The table includes different numbers of miRNA that were identified in this study.

Supporting Information Table S5. Calculation of Jaccard Similarity Index in different comparisons

Supporting Information Table S6. Differentially expressed miRNAs in different comparisons.

Supporting Information Table S7. List of miRNAs which expression either increases or decreases as altitude increases.

Supporting Information Table S8. ANOVA with miRNA expression was performed to find the significance of miRNA expression in the three populations of *Arabidopsis thaliana* grown in the two environmental conditions independently. Significant trait differentiation values are in italics ($P < 0.05$).

Supporting Information Table S9. The target of high altitude associated miRNAs and their differential expression.

Supporting Information Table S10. Correlation between miRNA expression bioclimatic variables. Correlation between miRNA expression and average temperature (AT), average precipitation (AP) and average radiation (AR) of growing season was calculated (r-values larger than the threshold (0.98) and P-value > 0.05).

Supporting Information Table S11. Putative new miRNAs predicted in this study by miRCat algorithms. The detailed parameter of predicted novel miRNA like their free energy, precursor length, abundance, sequence and genomic location are provided.

Supporting Information Table S12. The table includes expression abundant of novel miRNA and Indian *Arabidopsis*-specific novel miRNA.

Supporting Information Table S13. Two-way ANOVA was performed on plant morphology of UV-B treated plants. Two-way ANOVA was performed with population, UV treatment and their interaction as factors.
